# Sintilimab, stereotactic body radiotherapy and granulocyte–macrophage colony stimulating factor as second-line therapy for advanced non-small cell lung cancer: safety run-in results of a multicenter, single-arm, phase II trial

**DOI:** 10.1186/s13014-021-01905-3

**Published:** 2021-09-15

**Authors:** Jianjiao Ni, Yue Zhou, Lin Wu, Xinghao Ai, Xiaorong Dong, Qian Chu, Chengbo Han, Xiaofei Wang, Zhengfei Zhu

**Affiliations:** 1 Department of Radiation Oncology, Fudan University Shanghai Cancer Center, 270 Dong An Road, Shanghai, 200032 China; 2 Department of Oncology, Shanghai Medical College, Fudan University, Shanghai, China; 3 The Second Department of Thoracic Oncology, Hunan Cancer Hospital, The Affiliated Cancer Hospital of Xiangya School of Medicine, Central South University, Changsha, China; 4 Shanghai Lung Cancer Center, Shanghai Chest Hospital, Shanghai Jiao Tong University, Shanghai, China; 5 Cancer Center, Union Hospital, Tongji Medical School, Huazhong University of Science and Technology, Wuhan, China; 6 Department of Oncology, Tongji Hospital, Tongji Medical College, Huazhong University of Science and Technology, Wuhan, China; 7 Department of Oncology, Shengjing Hospital of China Medical University, Shenyang, China; 8 Department of Biostatistics and Bioinformatics, Duke University School of Medicine, Durham, USA; 9 Institute of Thoracic Oncology, Fudan University, 270 Dong An Road, Shanghai, 200032 China

**Keywords:** Sintilimab, Stereotactic body radiotherapy (SBRT), Granulocyte–macrophage colony stimulating factor (GM-CSF), Non-small cell lung cancer (NSCLC), Treatment-related adverse event (TRAE)

## Abstract

**Objectives:**

The SWORD trial is the first multicenter, single arm, phase II study assessing the safety and efficacy of a PD-1 inhibitor (Sintilimab), stereotactic body radiotherapy (SBRT) and granulocyte–macrophage colony stimulating factor (GM-CSF) in advanced non-small cell lung cancer (NSCLC) without sensitizing driver mutations. A safety run-in phase was conducted to determine the tolerability of the experimental treatment.

**Materials and methods:**

Twenty metastatic NSCLC patients who failed first-line chemotherapy were enrolled, and they received SBRT (8 Gy × 3) to one lesion, followed by Sintilimab (200 mg d1, every 3 weeks, until disease progression, unacceptable toxicity, or up to 35 cycles) and GM-CSF (125 μg/m^2^ d1-d14, cycle 1) within 2 weeks after SBRT. In addition, blood and tissue samples were serially collected for translational research.

**Results:**

Median age of the patients was 61 and all of them had more than 5 lesions at baseline. The sites of SBRT included lung (n = 11), mediastinal lymph node (n = 5), liver (n = 1), abdominal lymph node (n = 1), pleural nodule (n = 1) and vertebra (n = 1). No patients had dose-limiting toxicities (DLTs) and 18 patients experienced treatment-related adverse event (TRAE). The most common TRAEs were fatigue (50%), fever (30%), and ostealgia (20%), and they all were grade 1. Only 2 grade 3 TRAEs were observed, including elevation of liver enzymes in one and transient acute heart failure in another. No grade 4 or 5 AE was observed.

**Conclusion:**

Sintilimab, SBRT and GM-CSF for advanced NSCLC is safe with manageable TRAEs and the trial continues to recruit participants.

*Trial registration* ClinicalTrials.gov, NCT04106180. Registered 26 September 2019, SBRT in Combination With Sintilimab and GM-CSF for the Treatment of Advanced NSCLC-Tabular View-ClinicalTrials.gov.

**Supplementary Information:**

The online version contains supplementary material available at 10.1186/s13014-021-01905-3.

## Introduction

Over the last decade, immune checkpoint inhibitors, particularly inhibitors of the programmed cell death-1(PD-1)/programmed cell death ligand-1(PD-L1) axis, have transformed the therapeutic landscape in advanced non-small cell lung cancer (NSCLC) without driver mutations. Several PD-1/PD-L1 inhibitors have been demonstrated to provide significant overall survival (OS) benefit than docetaxel as second-line therapy [1–6]. However, the treatment efficacy of single-agent PD-1/PD-L1 inhibitors, which have an overall response rate (ORR) of around 15%–20% in all comers, is unsatisfactory [7].

Radiotherapy, especially stereotactic body radiotherapy (SBRT), is repeatedly found to enhance anti-tumor immunity and has the potential to synergize with immunotherapy in NSCLC [8, 9]. The underlying molecular mechanisms include the induction of immunogenic cell death [10], release of tumour associated antigen (TAA) and cytokines [11–13], and enhance the homing of immune cells to tumours [14, 15], thus converting immunologically “cold” tumours into “hot” tumours [16]. In addition, the upregulation of PD-L1 expression on tumour cells induced by radiotherapy makes patients more susceptible to subsequent PD-1/PD-L1 inhibitors, contributing to a higher response rate and longer survival [17]. Given that the radiation-induced antitumour immunity is dose-dependent, SBRT, which delivers a high radiation dose in generally 3–5 fractions to single-tumour sites with high accuracy, potentially has more potent immune activation effects than conventional radiotherapy [18, 19]. This superiority makes SBRT favorably combine with PD-1/PD-L1 inhibitors. In the phase II randomised clinical trial PEMBRO-RT, the combination of SBRT (8 Gy $\times$ 3 fractions) and Pembrolizumab revealed enhanced antitumour immunity with numerically improved ORR (36% vs. 18%), progression-free survival (PFS, 6.6 months vs. 1.9 months) and OS (15.9 months vs. 7.6 months) compared with Pembrolizumab alone [20]. Moreover, an individual patient-level meta-analysis of the Pembro-RT trial and MDACC study [21], demonstrated that adding radiotherapy, especially SBRT, to Pembrolizumab, significantly improved out-of-field (abscopal) response rate (ASR, 41.7% vs 19.7%, *p* = 0.0039), PFS (9.0 months vs 4.4 months, *p* = 0.0450) and OS (19.2 months vs 8.7 months, *p* = 0.0004) in patients with pretreated metastatic NSCLC [22]. However, these results need to be verified in further clinical trials enrolling patients from different races and genetic backgrounds. The reported efficacy of combining SBRT and PD-1/PD-L1 inhibitor remains unsatisfactory, and novel partners with non-redundant molecular mechanisms are demanded.

The antigen presentation by dendritic cells (DCs) and subsequent activation of adaptive immune response are indispensable steps in cancer-immune cycle, and the granulocyte–macrophage colony–stimulating factor (GM-CSF), which plays a pivotal role in the differentiation and maturation of DCs, can serve as potent immune adjuvant or sensitizer [23, 24]. In solid tumors, GM-CSF augments the recruitment and activations of DCs, which helps in eradicating cancer by presenting TAAs to T cells and subsequently initiating the anti-tumor adaptive immune response. This effect is supported by the GM-CSF-induced enhanced antitumour activity, which disappeared when CD4^+^ or CD8^+^ T cells were depleted [25]. In a preclinical study that used the B16 melanoma model, irradiated tumour cells alone could not stimulate significant anti-tumour immunity, whereas irradiated cells expressing murine GM-CSF stimulated potent, long-lasting, and specific anti-tumour immunity [25]. A proof-of-concept phase II trial (NCT02474186) enrolling 41 patients with different metastatic solid tumours found that adding GM-CSF to SBRT could induce abscopal response in 11 (26.8%) patients, including 4 patients with advanced NSCLC [26]. The safety and efficacy of combining radiotherapy and GM-CSF was shown in a phase I/II study in untreated stage III/IV squamous cell cancer of head and neck [27]. These data support the synergic cooperation between radiotherapy and GM-CSF in activating the innate immune response against cancer. However, the T cell exhaustion remains an obstacle for long-term anti-tumour immunity, which may be mitigated by PD-1/PD-L1 inhibitors. Hence, the triple combination of SBRT, GM-CSF and a PD-1/PD-L1 inhibitor may potentially enhance the treatment efficacy of advanced NSCLC through the activation of innate and adaptive anti-tumour immune responses. Nevertheless, feasibility and efficacy have not been evaluated.

Given the above preclinical and clinical data, we conducted a prospective, multicentre, single-arm, phase II trial assessing the safety and efficacy of triple combination of Sintilimab, a PD-1 inhibitor which had been proven to be effective in advanced NSCLC [28, 29], SBRT and GM-CSF as second-line therapy in sensitizing the driver mutation negative metastatic NSCLC. A safety run-in phase is conducted to determine the tolerability of this novel triple combination therapy by monitoring dose-limiting toxicities (DLTs) in the first 20 enrolled patients and herein we reported the results.

## Methods

### Study design

**Fig. 1:**
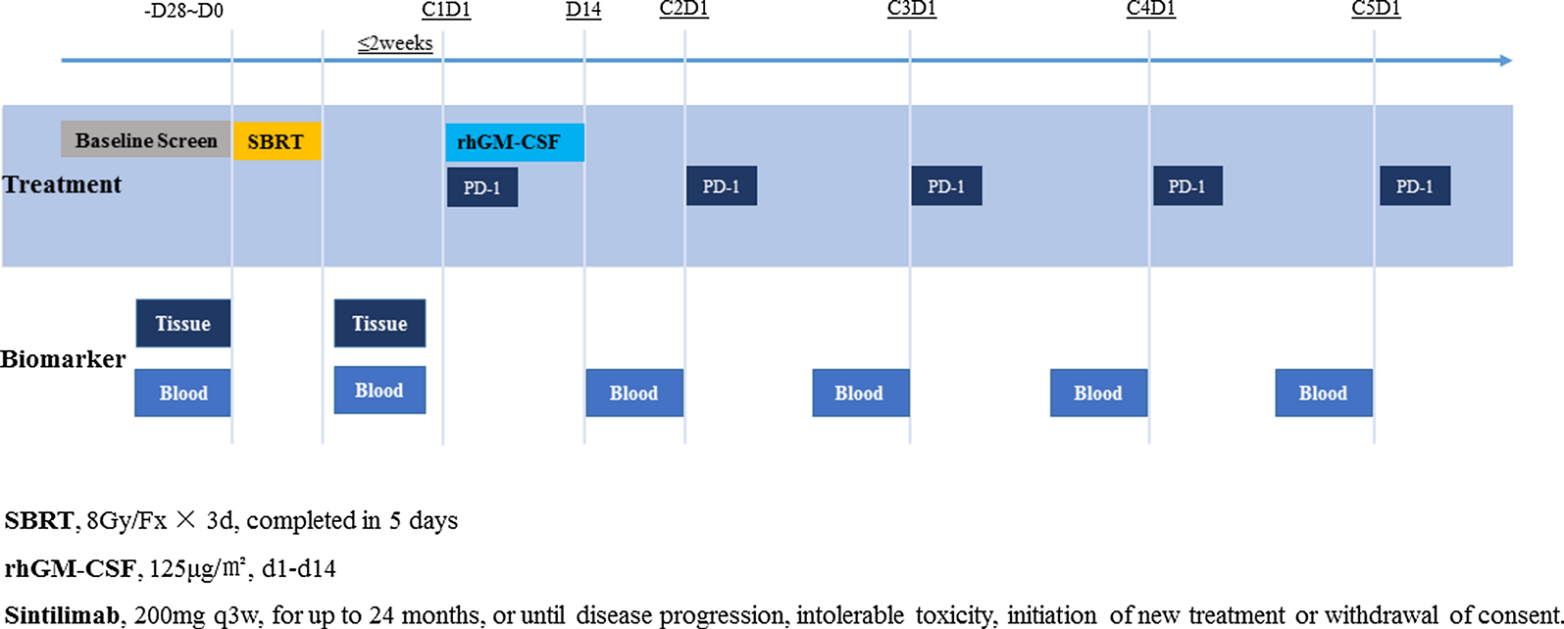
The study schema. Description: After baseline screen, eligible patients were treated with SBRT administrated at 24 Gy in 3 fractions (8 Gy/Fx) once-daily. Then, patients received Sintilimab, a PD-1 inhibitor, and rhGM-CSF starting within 2 weeks after SBRT completion. Sintilimab 200 mg was administrated on Day 1 (D1) every 3 weeks until disease progression, intolerable toxicity, up to 35 cycles (2 years), death, or withdrawal of consent. RhGM-CSF 125 μg/m2 would be given daily for 14 consecutive days (D1–D14) on Cycle 1. Treatment visits were performed with a physical examination, laboratory assessments, adverse events collections, and concomitant treatments description on D1 of each treatment cycle. Treatment efficacy was evaluated by radiographic examinations every 9 weeks. Abbreviations: SBRT: stereotactic body radiotherapy, PD-1: programmed cell death-1, rhGM-CSF: recombinant human granulocyte–macrophage colony stimulating factor

The SWORD trial (NCT04106180) was a single arm, open-label, multicentre, phase II study with a safety run-in phase. Patients with metastatic NSCLC and without driver mutations who failed first-line standard-of-care chemotherapy were treated with SBRT in combination with Sintilimab and GM-CSF. Additionally, blood and tissue samples were serially collected for translational research. The trial was designed to enrol 56 patients. The sample size was calculated for the following hypothesis: H_0_ (null) with an ORR ≤ 20% in accordance with Checkmate 017 and Checkmate 057 [5], H_1_ (alternative) with an ORR ≥ 38%. If no less than 17 out of the 56 patients evaluated had objective response (CR or PR), then H_0_ will be rejected in favour of H_1_. This design had at least 90% power to reject H_0_ if the ORR was 30% or more with a one-sided type I error rate of 5%. The study scheme is presented in Fig. 1 and the protocol of this trial is provided as Additional file 1.

### Objectives and endpoints

The trial was designed to evaluate the safety and efficacy of Sintilimab in combination with SBRT and GM-CSF in patients with advanced NSCLC previously treated with first-line platinum-based chemotherapy. The primary endpoint was ORR, which was defined as the proportion of participants with partial (PR) or complete (CR) response in evaluable patients in accordance with the Response Evaluation Criteria in Solid Tumors (RECIST) 1.1 [30] determined by investigators.

Secondary objectives were safety profiles according to the Common Terminology Criteria for Adverse Events version 5.0 (CTCAE 5.0), ASR, OS and PFS. ASR was defined as the proportion of patients with at least 30% reduction from baseline in the sum of longest diameter of non-irradiated target lesions defined by the RECIST 1.1 [30]. OS was defined as the time from the date of enrollment until death by any cause. Participants still alive at the time of last follow-up were censored in survival analyses. PFS was measured from the date of enrollment to the date of disease progression (PD) as defined by the RECIST 1.1 [30] or death due to any cause, whichever occurred first. Participants who did not have disease progression by the time of the last radiographic follow-up were censored in survival analyses.

Additionally, this study collected serial peripheral blood and tissue specimens from the enrolled patients who were willing to participate in the translational research. The relationship between biomarkers generated from these serially collected biological samples, treatment efficacy and treatment-related adverse events (TRAEs) were extensively analysed to investigate the molecular mechanisms underlying the synergic effect and primary resistance of this triple combination.

### Study population

Patients could be included if they had histologically confirmed stage IV NSCLC without driver mutations, and had failed (documented progression or intolerable toxicity) first-line platinum-based therapy without PD-1/PD-L1 inhibitors. Driver mutations included epidermal growth factor receptor mutations, anaplastic lymphoma kinase or ROS proto-oncogene 1 translocations. Patients should have at least one lesion (size: 1–5 cm) eligible for SBRT (24 Gy/3 Fx) and at least another measurable lesion as defined by the RECIST 1.1 [30]. Patients with brain metastasis were eligible if they were asymptomatic, neurologically stable, and off corticosteroids. Key exclusion criteria included patients who had received any PD-1/PD-L1 inhibitors previously.

### Treatment scheme and modalities

Eligible patients were treated with SBRT for one previously unirradiated primary or metastatic lesion (size 1–5 cm), and SBRT was administrated 24 Gy in 3 fractions (8 Gy/Fx) once daily for three consecutive days. Patients then received Sintilimab and GM-CSF intravenously (IV) starting within two weeks after SBRT completion. Sintilimab 200 mg IV was administrated on Day 1 (D1) every three weeks until PD, intolerable toxicity, up to 35 cycles (two years), death, or withdrawal of consent. GM-CSF 125 μg/m^2^ was given daily for 14 consecutive days (D1–D14) on cycle 1.

### Treatment evaluation

For patients who received at least one dose of Sintilimab and GM-CSF, treatment visits were performed with a physical examination, laboratory assessments, adverse event (AE) collections and concomitant treatments description on D1 of each treatment cycle. In addition, weekly phone calls were made to assess patients’ symptoms. Treatment efficacy was evaluated by radiographic examinations every nine weeks.

### Safety run-in analysis

Given that the safety profile of SBRT in combination with Sintilimab and GM-CSF has not been evaluated in patients with advanced NSCLC, a safety run-in was adopted to ensure that no excessive severe AE happened in treated patients. Accrual was suspended after the inclusion of 20 patients, and the safety data for each patient were collected within the first 30 days after the first dose of study drugs to detect unexpected non-tolerable toxicities early. DLT was monitored during the DLT observation window, which was defined as 30 days after the first cycle of protocol treatment. DLT was defined as any grade 3 event lasting for more than seven days other than asymptomatic laboratory abnormalities, any grade 4 event or any treatment related grade 5 event.

Grading was made in accordance with CTCAE 5.0. At any time during the safety run-in phase, if excessive numbers (≥ 35%) of DLTs were to be seen, the accrual was to be halted and the Data and Safety Monitoring Board was to be convened to decide on the study continuation. In addition, if excessive numbers (≥ 20%) of death were to be seen within 60 days since the initiation of GM-CSF treatment, the study was to close for safety concerns.

### Ethical considerations

Written informed consent was obtained from all patients before performing any study-specific tests or evaluations. The protocol received formal approval by the ethical authorities/committees of all participating academic cemtres.

### Statistical analysis

TRAEs and DLTs for the first 20 patients enrolled to the safety run-in phase of the trial were summarised by TRAE type, grade, frequency and proportion. These 20 patients were assessed and included for all outcomes by using the same schedule and criteria as subsequent patients. The demographic variables of these patients were summarised by median and range for continuous variables and by frequency and percentage for categorical variables. Analyses were performed by statisticians through the SPSS 21.0 (SPSS, Chicago, IL, USA) and R version 3.5.1 (The R Foundation for Statistical Computing).

## Results

### Patient characteristics

From 2019/10/16 to 2020/8/8, 22 patients were screened. Two patients were excluded due to withdrawal of consent in one patient and rapid deterioration of general condition before any treatment initiation in another. Hence, 20 patients were enrolled in the safety run-in phase. The baseline characteristics and SBRT sites of these 20 patients are summarised in Table 1. Most patients were male, smoker and had Eastern Cooperative Oncology Group (ECOG) performance status 1 and non-squamous NSCLC, with a median age of 61 years (range, 32–71 years). Baseline brain, liver and bone metastasis were present in 2, 4 and 8 patients, respectively. The majority of patients had no less than three metastatic organs and all patients had more than five metastatic lesions.

**Table 1:** Patient characteristics

Characteristics	Number of patients	%
Age		
≤ 60	8	40.0
> 60	12	60.0
Sex		
Female	5	25.0
Male	15	75.0
Smoking status		
Current/former	13	65.0
Never smoker	7	35.0
ECOG score		
0	2	10.0
1	18	90.0
Histology		
Squamous	8	40.0
Non-squamous	12	60.0
Metastasis at baseline		
Brain	2	10.0
Liver	4	20.0
Bone	8	40.0
No. of metastatic organs		
< 3	5	25.0
≥ 3	15	75.0
No. of metastatic lesions		
≤ 5	0	0.0
> 5	20	100.0
Site of SBRT		
Lung	11	55.0
Mediastinal LN	5	25.0
Abdominal LN	1	5.0
Liver	1	5.0
Thoracic vertebra	1	5.0
Pleural nodule	1	5.0

*ECOG* Eastern Cooperative Oncology Group, *SCC* squamous cell carcinoma, *NSCLC* non-small cell lung cancer, *SBRT* stereotactic body radiotherapy, *LN* lymph node

### AEs and DLT observation

No DLT was observed during the DLT observation window. Overall, 18 (90.0%) of 20 patients experienced TRAEs. The most common TRAEs were fatigue (50%), fever (30%), and ostealgia (20%), and all were grade 1. Grade 2 TRAEs occurred in four (40.0%) patients, whereas grade 3 TRAEs (ALT/AST elevation and heart failure) were observed in two (10.0%) patients. No patient experienced grade 4 or 5 TRAEs. The detailed information on AEs is summarised in Table 2.

**Table 2:** Treatment-related adverse events

	Grade 1	Grade 2	Grade 3	Grade 4
Fever	6 (30.0%)	0	0	0
Fatigue	10 (50.0%)	0	0	0
Headache	3 (15.0%)	0	0	0
Ostealgia	4 (20.0%)	0	0	0
Skin rash	2 (10.0%)	0	0	0
Pruritus	2 (10.0%)	0	0	0
Elevation in ALT	0	1 (5.0%)	1 (5.0%)	0
Elevation in AST	0	1 (5.0%)	1 (5.0%)	0
Decreased appetite	2 (10.0%)	0	0	0
Nausea	2 (10.0%)			
Diarrhea	2 (10.0%)	0	0	0
Cough	1 (10.0%)	0	0	0
Vomiting	1 (10.0%)	0	0	0
Chest pain	0	1 (5.0%)	0	0
Hypothyroidism	0	1 (5.0%)	0	0
Heart failure	0	1 (5.0%)	1 (5.0%)	0

*ALT* alanine aminotransferase, *AST* aspartate aminotransferase

### Characteristics of patients experiencing acute heart failure

Two patients experienced acute and transient left heart failure.

Patient 009 was a 62-year-old male, a smoker, and diagnosed with metastatic squamous cell carcinoma of the right lung in July 2019. He denied any previous medical history of cardiovascular diseases. After failing first-line chemotherapy consisting of four cycles of cisplatin and nab-paclitaxel, he was enrolled into the trial. SBRT was performed to one of the metastatic mediastinal lymph nodes uneventfully, followed by Sintilimab and GM-CSF. Four days after Sintilimab and GM-CSF initiation, the patient complained of progressive dyspnoea with moist rales in the lungs. Laboratory tests revealed an elevation in pro-BNP (3970 pg/ml) and echocardiography found mild pulmonary hypertension. Acute left heart failure (grade 2) was diagnosed by an experienced cardiologist and GM-CSF was stopped. The patient recovered three days after administration with diuretics, oxygen therapy and antiasthmatic agents. However, the patient refused to continue the study treatment and was transferred to a local hospital.

Patient 014 was a 71-year-old male, smoker, and diagnosed with metastatic squamous cell carcinoma of the right lung in September 2019. He also denied any past medical history of cardiovascular diseases. Before enrollment, he had been treated with four cycles of Carboplatin and Gemcitabine, as well as salvage thoracic radiotherapy. SBRT was performed to one of the metastatic mediastinal lymph nodes successfully, followed by Sintilimab and GM-CSF. Seven days after Sintilimab and GM-CSF initiation, the patient developed severe shortness of breath, fatigue and facial oedema. On examination, moist rales were observed in the lungs, and the oxygen saturation was 86%. Laboratory tests found a significant elevation in pro-BNP (4820 pg/ml). Acute left heart failure (grade 3) was diagnosed, and GM-CSF was stopped. The patient’s symptoms were alleviated five days after the administration of diuretics and oxygen therapy. This event was considered related to GM-CSF only. Thus, Sintilimab was given for another three cycles, without the recurrence of acute heart failure. The best response to the triple therapy was a stable disease. However, the tumour progressed rapidly after the fourth cycle and eventually led to death.

## Discussion

PD-1/PD-L1 inhibitors, alone or in combination with other agents, are now standard of care for advanced NSCLC without driver mutations [31]. However, the response rate of PD-1/PD-L1 inhibitor alone is limited and the treatment toxicities of combinational modalities already available are frequent [32, 33]. Novel treatment strategies with potent efficacy and minimal AEs are highly needed. Here, we report the preliminary results of safety run-in phase of the prospective, multicentre, phase II trial (SWORD) investigating triple combination of Sintilimab, SBRT and GM-CSF as novel second-line therapy for advanced NSCLC without driver mutations. The novel combination is safe with manageable TRAEs.

For the first time, we have found that the triple combination of SBRT, a PD-1 inhibitor and GM-CSF seems to be safe with mild toxicities. The safety of PD-1/PD-L1 inhibitors in combination with SBRT has been demonstrated in several prospective clinical trials [20, 21, 34, 35], with the frequency of grades 3–5 TRAEs ranging from 9.7% to 30.0% and very few patients developed DLTs. The combination of SBRT and GM-CSF has also been tested in a prospective trial with acceptable toxicities [26]. However, the feasibility and safety of combining GM-CSF and PD-1/PD-L1 inhibitors have not been reported. In our study, grade 3 TRAEs occurred in 10.0% of the patients, without DLT or grade 4–5 TRAEs. Most of the TRAEs were mild, and the two grade 3 TRAEs were transient, which could be successfully managed. Taken together, triple combination of Sintilimab, SBRT and GM-CSF in advanced NSCLC was demonstrated to be safe, and the trial has continued to recruit participants.

Nevertheless, acute heart failure was an unexpected AE in our study. Congestive heart failure was previously reported in clinical trials examining the safety and efficacy of combinational regimens involving GM-CSF amongst patients with haematological disorders [36, 37] and solid tumours [38]. The GM-CSF receptor expression and plasma GM-CSF level were also found to be significantly elevated in patients with end-stage heart failure [39, 40]. Mechanically, GM-CSF produced by cardiac fibroblasts could act locally and distally to generate and recruit inflammatory and proteolytic cells, which led to heart failure in mouse models [41]. In our study, two patients experienced transient acute left heart failure and recovered quickly after GM-CSF discontinuation and initiation of diuretics. Hence, GM-CSF-related heart failure was suspected in these two patients. However, we could not totally rule out the possibility that Sintilimab also had a role in the development of acute heart failure in these two patients. The cardiac toxicities of PD-1/PD-L1 inhibitors rarely developed but have detrimental effects [42, 43], and PD-1/PD-L1 inhibitor-related heart failure was anecdotally reported [44–46]. However, the two patients recovered rapidly without glucocorticoids, and one of the patients continued to receive further cycles of Sintilimab without the recurrence of heart failure. Hence, acute heart failure should be intensively monitored in patients receiving continuous GM-CSF especially amongst those with concurrent PD-1/PD-L1 inhibitors.

Our study has several strengths. Firstly, this prospective, phase II, multicentre study is the first to assess the safety and efficacy of a triple combination of a PD-1/PD-L1 inhibitor, SBRT and GM-CSF in advanced solid tumors. Although this study is a single-arm study, several clinical trials test the efficacy of PD-1/PD-L1 inhibitor alone as second-line therapy in advanced NSCLC with generally consistent results, which can serve as reliable historical control [1–3, 47]. These historical controls and experiences from previous studies are used [20, 21], and the sample size of our study is dedicatedly designed to have a 90% power to detect the difference. Moreover, the data generated from serially collected biological samples can provide valuable information regarding the molecular mechanisms underlying the effective anti-tumor immune response induced by the trial regimen, which can be used to design improved combinational treatment strategies for advanced NSCLC. Notably, the effect of SBRT on the immune microenvironment of tumour lesions outside the radiation field and its relationship between the so-called “abscopal effect” can be examined, which is largely unknown, because tissue samples from the same tumor lesion outside the radiation field both before and after SBRT (but before initiation of Sintilimab and GM-CSF) are collected [48, 49].

## Conclusions

The triple combination of SBRT, GM-CSF and Sintilimab is safe and well tolerated. The SWORD trial continues patient recruitment and the efficacy results are pending.

## Supplementary Information

**Additional file 1**. A phase II, open-label, single-arm, multi-center study of Sintilimab, stereotactic body radiotherapy and granulocyte-macrophage colony stimulating factor in advanced non-small cell lung cancer (SWORD).

## Data Availability

The datasets used and/or analysed during the current study are available from the corresponding author on reasonable request.

## Abbreviations

PD-1: Programmed cell death-1
PD-L1: Programmed cell death ligand-1
NSCLC: Non-small cell lung cancer
OS: Overall survival
ORR: Overall response rate
SBRT: Stereotactic body radiotherapy
TAA: Tumor associated antigen
PFS: Progression-free survival
ASR: Abscopal response rate
DC: Dendritic cell
GM-CSF: Granulocyte–macrophage colony stimulating factor
DLT: Dose-limiting toxicity
PR: Partial response
CR: Complete response
RECIST: Response Evaluation Criteria in Solid Tumors
CTCAE 5.0: Common Terminology Criteria for Adverse Events version 5.0
PD: Progression disease
TRAE: Treatment-related adverse events
D: Day
AE: Adverse event
ECOG: Eastern Cooperative Oncology Group
