# Supplementary material for: Sintilimab, stereotactic body radiotherapy and granulocyte–macrophage colony stimulating factor as second-line therapy for advanced non-small cell lung cancer: safety run-in results of a multicenter, single-arm, phase II trial
Source: Radiat Oncol. 2021 Sep 15;16:177. doi: 10.1186/s13014-021-01905-3 (PMC8444553; doi:10.1186/s13014-021-01905-3)
Supplement: Supplementary file 1 — Additional file 1. A phase II, open-label, single-arm, multi-center study of Sintilimab, stereotactic body radiotherapy and granulocyte-macrophage colony stimulating factor in advanced non-small cell lung cancer (SWORD). [file 13014_2021_1905_MOESM1_ESM.docx]

**A phase II, open-label, single-arm, multi-center study of Sintilimab, stereotactic body radiotherapy and granulocyte-macrophage colony stimulating factor in advanced non-small cell lung cancer (SWORD)**

**Protocol version1.2**

(2019.06.12)

1. Background

# Lung cancer is one of the most common cancers worldwide and there are approximately 1.8 million new cases by 2012, representing 12.9% of all new cancers^[1]^. It is also the most common cause of deaths from cancer, accounting for 19.4% of all cancer-attributed deaths^[2]^. Non-small cell lung cancer (NSCLC) represents 80% to 85% of lung cancers. Unfortunately, approximately 50% of NSCLC patients already have advanced disease at the time of diagnosis. Treatment options for advanced NSCLC patients are very limited after the failure of first-line therapy and the prognosis of these patients is dismal, with a 5-year overall survival (OS) rate less than 10%^[4, 5]^.

Especially, advanced NSCLC patients without driver mutations have few effective drugs. Targeted therapy has significantly improved the survival of metastatic NSCLC patients with driver mutations, such as epidermal growth factor receptor (EGFR) mutations, anaplastic lymphoma kinase (ALK) translocations and ROS proto-oncogene 1 (ROS1) translocations. Tyrosine kinase inhibitors (TKIs) have been established as standard first-line treatments for these patients with driver mutations, with a median OS of more than 30 months^[6]^. However, treatments for advanced NSCLC without driver mutations remain an urgent unmet need. For these patients, the median OS is about 11 months. Even with maintenance chemotherapy, it is only increased by about 2 months^[7]^. Since the majority advanced NSCLC patients have no targetable driver mutations, it is of great clinical significance to improve both the survival and quality of life in these patients.

As early as 1953, Mole et al. observed the regression of tumor outside the irradiated field, and called it the abscopal effect^[8]^. Recently, the mechanisms underlying the immune-regulatory effects of radiotherapy have been illucidated. Radiotherapy induces immunogenic cell death (ICD) of tumor cells and causes the release of tumor associated antigens, dAMP and cytokines, which stimulates the maturation of dendritic cells (DCs). Mature DCs present tumor antigens to T cells. Then T cells are activated and expanded into tumor-specific T cells, thereby producing anti-tumor immune effects^[9]^. Radiotherapy also induces the up-regulation of major histocompatibility complex-I (MHC-I), which plays a crucial role in the activation of T cells^[10]^. Radiotherapy can also induce the up-regulation of tumor-associated antigens on the tumor cell membrane, thus enabling the immune system to recognize tumor cells more effectively^[9]^. In addition, radiotherapy alters the MHC-I-associated peptide profiles, some of which can only be induced specifically by radiation, recognized as "foreign peptide"(neoantigen) by the immune system^[10]^. These mechanisms lead to the use of radiotherapy as an *in-situ* tumor vaccine, which stimulates specific anti-tumor immunity to kill tumors outside the irradiated field. However, radiotherapy using conventional fractionation is not potent enough to stimulate sufficient immune cells to infiltrate the tumor sites. As the body has an effective set of immunosuppressive mechanism to prevent autoimmunity, it also greatly limits the anti-tumor immune effect, which partially explains why the abscopal effect is rare when radiotherapy of conventional fractionation is used alone^[11]^.

Stereotactic Body Radiation Therapy (SBRT) is a technique for delivering high doses of radiation to tumors while preserving the normal tissues located around this area. In addition to effectively killing tumor cells in the irradiated field, SBRT can also mediate immune response and cause the regression of un-irradiated lesions. SBRT increases the fractional dose and reduces the number of exposures. It also has higher conformability to the tumor and less damage to the surrounding tissues. Its larger local dose causes the irradiated tumor cells to release more tumor-associated antigens. Meanwhile, the lymphatic tissues around the tumor get better protection. These makes SBRT more effective in inducing anti-tumor immunity. The concept of iSABR has been put forward in 2016, which suggests that SABR can stimulate the immune response more effectively than conventional fractional radiotherapy based on preclinical evidence and clinical data, and points out the important research value of the combination of iSABR with other therapies^[12]^. In a phase I study of ipilimumab combined with SBRT (single dose 6Gy-12.5Gy, total dose 50-60Gy) for malignant melanoma, 31 out of the 35 patients enrolled did not progress in the out-of-field lesions, including 3 (10%) partial response (PR), and 7 (23%) having clinical benefit [PR or stable disease (SD)] for more than 6 months^[13]^. A meta-analysis of 14 studies on the abscopal effects of radiotherapy in murine model showed that the incidence of abscopal effects increased as the biological equivalent dose (BED) of the irradiated tumor elevated^[14]^.

Immune checkpoint inhibition (ICI) has revolutionized the treatment landscape of advanced NSCLC. Programmed cell-death protein 1 (PD-1) is an immune checkpoint molecule. PD-L1 and PD-L2, the ligands of PD-1, are up-regulated in a variety of tumor cells and tumor infiltrating cells. The interaction of PD-1 and its ligands (mainly PD-L1) can lead to the inhibition of cytotoxic T cells and the enhancement of regulatory T cells (Tregs), thereby inhibiting anti-tumor immunity. Sintilimab, a fully humanized anti-PD-1 monoclonal antibody, can block the interaction between PD-1 and PD-L1/PD-L2, subsequently induce strong anti-tumor response, which has confirmed *in vitro* and *in vivo*, with comparable affinity, pharmacokinetic characteristics and safety to pembrolizumab and nivolumab. For NSCLC, PD-1 monoclonal antibody nivolumab and pembrolizumab, as well as PD-L1 monoclonal antibody, atezolizumab and durvalumab, have all shown significant survival benefits when compared with the current standard of care treatments^[17-21]^. In the Keynote001 study, the ORR of pembrolizumab was 24.8% in untreated patients and 18.0% in previously-treated patients^[21]^. In the Keynote010 study, 1034 previously-treated advanced NSCLC patients were enrolled and divided into docetaxel group, low-dose pembrolizumab group and high-dose pembrolizumab group. An improved OS was observed in the high-dose pembrolizumab group compared with the low-dose pembrolizumab group and the docetaxel group (12.7 months vs 10.4 months vs. 8.5 months)^[22]^. In the PACIFIC study, durvalumab was used for unresectable stage III NSCLC without progression after standard platinum-based concurrent chemoradiotherapy. Durvalumab maintenance significantly prolonged PFS and OS compared with placebo^[20]^. However, the treatment efficacy of PD-1/PD-L1 inhibitors alone in unselected advanced NSCLC remain limited, with an ORR around 15%-20%, highlighting the crucial needs for the development of novel combinational treatment strategies with higher potency and less toxicity.

Based on the above data, we hypothesized that sintilimab combined with radiotherapy may have a synergistic effect in promoting anti-tumor immune effect, which has been initially manifested in similar drugs. In a case report where a patient with stage IIIB NSCLC received SBRT in combination with nivolumab, the irradiated tumors, as well as the metastatic lymph nodes outside the irradiation field, all reached CR^[15]^. The abscopal effect was also observed in two patients with metastatic skin Merkel cell carcinoma who were treated with the combination of pembrolizumab and single-dose palliative radiotherapy^[16]^. However, the feasibility and efficacy of sintilimab and SBRT in advanced NSCLC remain undetermined.

Granulocyte-macrophage Colony Stimulating Factor (GM-CSF) is a hematopoietic growth factor that can promote the differentiation, maturation, and expansion of DCs and enhance their ability to recognize and process tumor antigens and present tumor antigens to T cells, thereby promoting anti-tumor immune responses. Mature DCs are critical in generating anti-tumor immune response. Compared with immature DCs, mature DCs have membrane receptors that mediate the uptake of antigens such as Fc receptors, giving these cells stronger capabilities of uptaking and processing antigens. Mature DCs also have a high level of MHC-II molecules, costimulatory molecules, and adhesion molecules, which improves their antigen presentation ability. Since GM-CSF promotes the maturation of DCs, it is theoretically effective in promoting anti-tumor immune response. Preclinical studies have found that the irradiated malignant melanoma cell is insufficient to produce anti-tumor immune effects, while the concurrent use of retrovirus which could secret GM-CSF enables the melanoma cell to produce strong and long-lasting anti-tumor immunity^[23]^. In a clinical study where 41 patients with metastatic malignant tumors were treated with radiotherapy combined with GM-CSF, abscopal effect were seen in 11 patients (26.8%), and it was particularly significant that 4 out of the 11 NSCLC cases had objective response out-of-field ( 2 CR at the remote sites). Furthermore, patients with abscopal effect was found to have longer OS. As no dose reduction or treatment interruption occurred due to treatment related toxicity, the combination of GM-CSF and radiotherapy is considered a safe treatment for inducing anti-tumor immune effects^[24]^. Another study enrolling 72 lung cancer and 54 esophageal cancer patients examined the serum levels of GM-CSF before and during radiotherapy, and found that patients with elevated level of serum GM-CSF after radiotherapy had significantly prolonged PFS and OS^[25]^. Taken together, we hypothesized that the combination of SBRT and GM-CSF may bring survival benefits in advanced NSCLC.

In summary, the combined application of SBRT, sintilimab, and GM-CSF may enhance the effect of immunotherapy, thereby improving the clinical efficacy in advanced NSCLC. So far, there have been no prospective studies to confirm this scientific hypothesis, as well as the clinical value of the combination therapy. Therefore, we plan to carry out a phase II study of SBRT combined with sintilimab and GM-CSF in the treatment of advanced NSCLC, attempting to explore the feasibility of this triple therapy which mechanically could enhance anti-tumor immune response through promotion of both innate and adaptive immune processes.

1. Study objectives

To explore the safety and preliminary efficacy of SBRT combined with sintilimab and GM-CSF in stage IV NSCLC patients without driver mutations such as EGFR, ALK, and ROS1.

**2.1 Primary objective:**

**Overall Response Rate (ORR):** Tumor response is evaluated by investigators according to the Response Evaluation Criteria in Solid Tumors (RECIST) 1.1. ORR is defined as the proportion of patients who experienced CR or PR as the best response in the total evaluable cases.

**2.2 Secondary objectives:**

**Treatment-related adverse events (TRAEs):** The proportion of the treatment-related toxic reactions in the total evaluable cases according to the Common Terminology Criteria Adverse Events (CTCAE) 5.0.

**Out-of-field (abscopal) response rate (ASR):** At any time through enrollment to last follow-up, the longest diameter of any un-irradiated target lesions (per RECIST v1.1) reduce ≥30% will be considered as out-of-field (abscopal) response. The ASR is defined as the proportion of patients with an out-of-field response in the total evaluable patients.

**Overall survival (OS):** The time interval from enrollment to death due to any cause. Patients who are still alive at the time of analysis will be censored at the date of last contact.

**Progression free survival (PFS):** The time interval from enrollment to the first documented disease progression or death due to any cause. Patients who are still alive without disease progression at the time of analysis will be censored at the last date of last radiographic follow-up.

**2.3 Exploratory objectives:**

Serial peripheral blood and/or tissue specimens will be collected from the enrolled patients who are willing to provide samples for exploratory research. The relationship between biomarkers generated from these serially collected biological samples and treatment efficacy, as well as TRAE, will be extensively investigated.

1. **Subject selection**

**3.1 Inclusion criteria:**

Subjects must meet all of the following inclusion criteria to participate in this study.

1. Age ≥ 18 years old.
2. ECOG performance status 0-1.
3. Histologic or cytologic confirmed NSCLC, stage IV (AJCC 8th).
4. Driver mutations (including EGFR, ALK, and ROS-1) negative.
5. Disease progressed after first-line platinum-based chemotherapy (excluding anti-PD-1/PD-L1 therapy).
6. Subjects with stable or asymptomatic brain metastases can be included, but they must have stable lesions and no neurological symptoms without receiving systemic glucocorticoid therapy.
7. At least 1 lesion with a longest diameter of 1-5cm which was suitable for SBRT at a dose of 24Gy/3Fx, and at least 1 evaluable (RECIST1.1) lesion other than the one to receive SBRT. Lymph nodes can be selected as independent evaluable lesions and SBRT lesions, while brain metastasis cannot be chosen as either SBRT or out-of-field evaluable lesions.
8. Previous radiotherapy is permitted as long as the following conditions were met: 1) SBRT and out-of-field evaluation evaluable lesions must have not received previous radiotherapy; 2) dose received at normal tissue will not be affected by the previous radiotherapy. (Note: If the subject has received previous radiotherapy, detailed radiotherapy-related parameter data should be provided).
9. No palliative radiotherapy was required during study period per investigators’ judgment.
10. Subject who has undergone surgery must fully recover from the toxicity and complications of the surgery before starting treatment.
11. Subjects are encouraged to provide serial blood and tissue samples before and during treatment per protocol requirements.
12. Males/females of child-bearing age should agree to contraception during the trial (surgical ligation or oral contraceptives/intrauterine contraceptives + condoms).
13. Life expectancy ≥ 3 months.
14. Organ function reached the following standards within one week before enrollment:
15. Bone marrow function: hemoglobin ≥ 80 g/L, white blood cell count ≥ 4.0 * 10^9/L or neutrophil count ≥ 1.5 * 10^9/L, platelet count ≥ 100 * 10^9/L;
16. Liver: Serum total bilirubin level ≤ 1.5 times the upper limit of normal (ULN), and aspartate aminotransferase (AST) and alanine aminotransferase (ALT) ≤ 2.5 times ULN. For serum total bilirubin level > 1.5 times ULN, the direct bilirubin level must ≤ ULN;
17. Kidney: serum creatinine level < 1.5 times ULN or creatinine clearance rate ≥ 50 ml/min, urea nitrogen ≤ 200 mg/L, serum albumin ≥ 30 g/L.
18. The subject must have the ability to understand and to voluntarily sign an informed consent form.

**3.2 Exclusion criteria:**

Subjects who meet any of the following exclusion criteria are not eligible to participate in this study:

1. The subject had received anti-PD-1/PD-L1 inhibitors or other immune checkpoint inhibitors.
2. The subject has severe autoimmune diseases: active inflammatory bowel disease (including Crohn’s disease and ulcerative colitis), rheumatoid arthritis, scleroderma, systemic lupus erythematosus, autoimmune vasculitis (such as Weiger Granuloma), etc..
3. The subject has symptomatic interstitial lung disease or active infectious/non-infectious pneumonia.
4. The subject has any risk factors for intestinal perforation: active diverticulitis, intra-abdominal abscess, gastrointestinal (GI) obstruction, abdominal cancer or other known risk factors for intestinal perforation;
5. The subject has a history of other malignant tumors;
6. The subject has active infection, heart failure, myocardial infarction, unstable angina or unstable arrhythmia within 6 months before enrollment;
7. The subject has physical examination or clinical findings, or other uncontrollable disease, which the investigator believes may interfere with the outcome or increase risk of treatment complications.
8. Palliative radiotherapy is required per investigator’s judgement.
9. The first-line treatment does not include platinum-based chemotherapy.
10. The tumor is mixed with components of small cell lung cancer;
11. Women who are breastfeeding or pregnant.
12. The subject has congenital or acquired immunodeficiency diseases including human immunodeficiency virus (HIV), or a history of organ transplant or allogeneic stem cell transplant.
13. The subject has known hepatitis B virus (HBV), hepatitis C virus (HCV) or active tuberculosis infection.
14. The subject has received oncological vaccines or other vaccines within 4 weeks before the start of treatment.

(Note: Seasonal influenza vaccines for injection are mostly inactivated and therefore are allowed, whereas intranasal vaccines are usually live attenuated vaccines and therefore are not allowed.)

1. The subject uses other immune-agents, chemotherapeutic agents, other drugs in clinical studies, and long-term cortisol during the study period.
2. The subject has mental illness, substance abuse, or social problems that affect compliance are not included after being reviewed by a doctor.
3. The subject is allergic or contraindicated to sintilimab or GM-CSF.

**3.3 Subject withdrawal/discontinuation criteria:**

1. Inappropriate enrollment. Those who have not received any treatment after enrollment should withdraw immediately, and the subject's information will not be included in the study analysis. If the subject has already received study treatment, the investigator will evaluate the subject and determine the benefit and risk of the subject before deciding whether to withdraw. If the subject needs to be excluded, he/she will be withdrawn from the study after the completion of follow-up after treatment. The subject’s information will not be included in the efficacy analysis of this study, but should be included in the safety analysis.
2. Any of the following situations occur during the pre-treatment evaluation stage: new or deteriorated systemic disease which meet the exclusion criteria, voluntarily withdraw consent or lost to follow-up or death; poor compliance.
3. For those who are considered to be excluded by investigators, the investigator shall report the reasons for the exclusion to the principal investigator. When permitted, patients who have not yet received any study treatment should be withdrawn immediately, and the information of this subject will not be included in the study analysis. If the subject has already received the protocol specified treatment, he/she will be withdrawn from this study after the completion of the follow-up after treatment, and the subject will not be included in the efficacy analysis of this study, but should be included in the safety analysis.
4. Subjects may request withdraw from the clinical trial at any time during the study period if they do not want to continue.
5. If serious adverse events occur, the subject might be withdrawn on the judgment of the investigator.
6. Deterioration during the trial, which may threaten the life of the subject, or other conditions that may affect the observation appear during the trial.
7. The subject lost follow-up or die during the treatment period.
8. The subject received chemotherapy, Chinese medicines, other immunotherapy drugs, radiotherapy sensitizers, or other drugs that affect the efficacy and toxicity evaluation during the trial.
9. Serious deviations which makes it difficult to evaluate the drug effect.
10. Poor compliance.
11. **Study plan and timing of procedures**


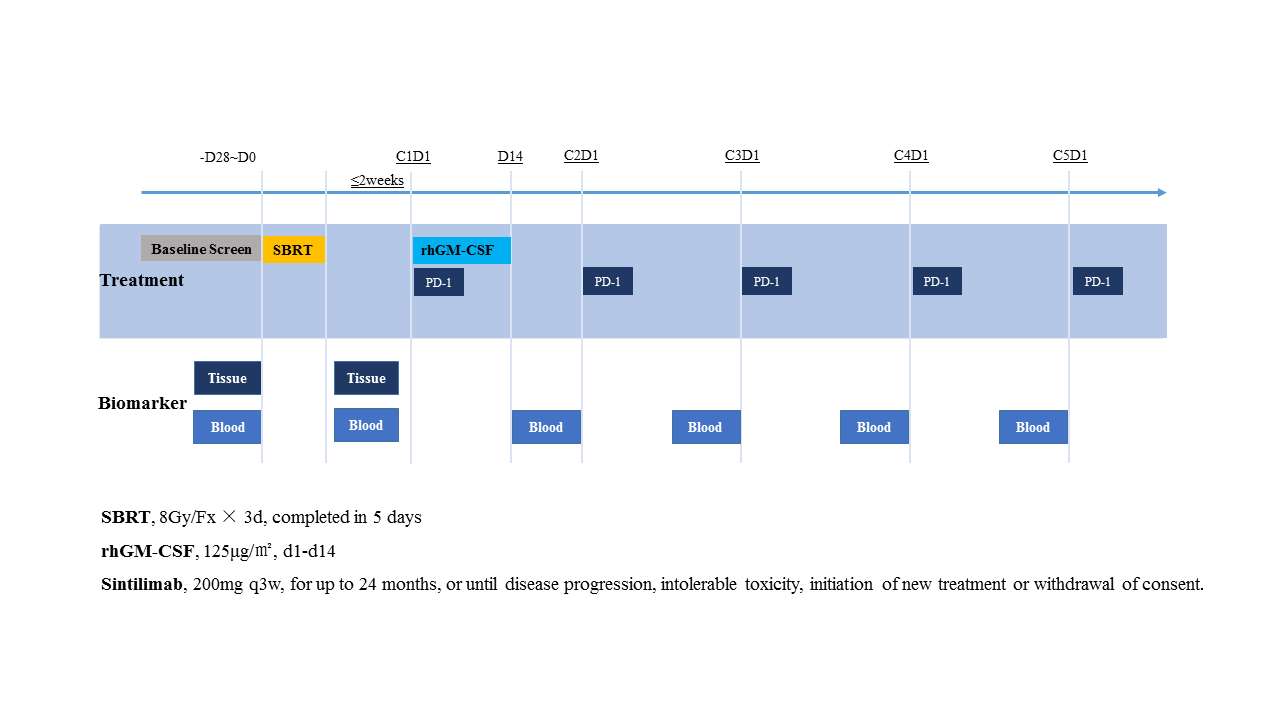


**Figure 1. Study flow**

**Table 1. Study schedule**

|  | **Baseline screening** | **SBRT** | **Sintilimab, GM-CSF treatment** | **Sintilimab maintenance** | | **End of treatment**  **Survival follow-up** |
| --- | --- | --- | --- | --- | --- | --- |
| Window (days) | Within 28 days before the start of SBRT | End of SBRT  (or before D1 administration) | C1D1  D5/9/14 (± 2 days) | C2D1  (±3 days) evaluation before each administration | CnD1  (±3 days) | Every 3 months  (±7 days) |
| Frequency |  |  | 3 times in total | Every 3 weeks | Every 9 weeks  (every 3courses of Sintilimab) | Every 3 months |
| **Research procedure** |  | | | | | |
| Informed consent^1^ | X |  |  |  |  |  |
| Inclusion criteria | X |  |  |  |  |  |
| Demography/ Medical history/ Previous treatments^2^ | X |  |  |  |  |  |
| Vital signs ^3^ |  |  |  |  |  |  |
| Weight/Height^4^ | X |  |  | X | X |  |
| Medical history^5^/ Physical examination | X |  | X | X | X | X |
| ECOG PS score | X |  |  | X | X | X |
| Blood routine ^6^ | X |  | X | X | X | X |
| Coagulation function ^7^ | X |  |  | X | X | X |
| Blood Biochemistry ^8^ | X |  | X | X | X | X |
| Thyroid function ^9^ | X |  |  | X | X | X |
| Pregnancy test ^10^ | X |  |  |  |  |  |
| Urinalysis ^11^ | X |  |  | X | X |  |
| Virological antibody testing (HIV, HBV and  HCV) | X |  |  |  |  |  |
| 12-lead ECG | X | when necessary | | | | |
| Lung function | X |  |  |  |  |  |
| Adverse event assessment ^12^ |  | X | X | X | X | X |
| Concomitant medication | X | X | X | X | X | X |
| Subsequent anti-tumor treatment |  |  |  |  |  | X |
| Telephone follow-up (when outpatient follow-up is not possible) |  |  |  |  |  | X |
| **Efficacy evaluation** |  | | | | | |
| Radiographic assessment ^13^ | X |  |  |  | X | X |
| Gene mutation detection ^14^ | X |  |  |  |  |  |
| **Biomarker exploration** |  | | | | | |
| Blood specimens for biomarker studies ^15^ | X | X (end of SBRT) | X | X | X | X |
| Tumor puncture ^16^ | X | X (end of SBRT)  (Recommended, not required) | X (after d14 GM-CSF medication,  Recommended, not required) |  |  |  |

1. The signing of the ICF should be performed before any study procedure.
2. Previous treatments include treatment for the initial diagnosis, including chemotherapy, radiotherapy, and surgical treatment, as well as treatment for previous co-existing diseases within the past 30 days.
3. Vital signs include temperature, pulse, respiratory rate, and blood pressure.
4. Height measurement is only performed during the screening period. Weight of subjects is measured before each administration. If the weight of the subject fluctuates less than 10% from the baseline (the day of the first study dose), the baseline weight is used to calculate the dose of chemotherapeutic drugs. Otherwise, the actual dose is calculated according to the body weight on the day of the planned administration.
5. Medical history must include medical and surgical history, history of smoking/alcohol consumption/family history, previous radiotherapy and chemotherapy, and symptoms of intracranial hypertension.
6. Blood routine includes red blood cell count (RBC), hemoglobin (Hb), white blood cell count (WBC), and white blood cell count (absolute neutrophil count and absolute lymphocyte count are required), hematocrit (HCT), platelets (PL).
7. Coagulation functions include PT and INR. It is carried out within 28 days before the first study dose during the screening period and during the safety follow-up. The lab tests are carried out in various research centers.
8. Blood biochemistry includes blood electrolytes (K, Na, Ca, Mg, Cl, HCO_3_^-^ are required), ALT, AST, γ-GT, total bilirubin, direct bilirubin, amylase, lipase, creatinine, urea nitrogen, serum albumin, blood glucose.
9. Thyroid function includes T3, T4, fT3, fT3, TSH.
10. Women of child-bearing age should undergo a urine or serum pregnancy test within 28 days before the first dose in the screening period. If the urine pregnancy test result cannot be confirmed as negative, a serum pregnancy test should be performed, and the serum pregnancy result shall prevail. Tests will be carried out in various research centers.
11. Urinalysis must include urine specific gravity (SG), urobilinogen (URO), occult blood (BLD), white blood cells (WBC), urine protein (PRO), urine glucose (GLU), bilirubin (BIL), ketone bodies (KET), urine red blood cells (RBC), urine color (COL).
12. The assessments of AE and laboratory test safety are evaluated according to CTCAE v 5.0. Definition, recording, relevance judgments, severity judgments, reporting time limits and management of AE and SAE are described in sections 5 and 7 of the protocol.
13. Imaging examinations at baseline screening include: Chest CT examination within 28 days before treatment. PET-CT can be used instead. Abdominal CT/MRI within 28 days before treatment. PET-CT can be used instead. Cranial MRI examination within 28 days before treatment. Patients with metal foreign bodies or implants that prevent them from receiving cranial MRI can receive enhanced skull CT instead. Whole-body bone scan within 28 days before treatment. Bone scan is excused if PET-CT shows negative bone metastases. PET-CT is not necessary. In the follow-up (once every 3 courses of Sintilimab), imaging examinations, except for the parts with local symptoms, only examine the location of the lesion at baseline, and the same imaging examination methods as the baseline should be used. The examination items for different sites are listed as follow: chest: chest CT, which can be replaced by PET-CT; abdomen: abdominal CT/MRI, which can be replaced by PET-CT; cranial MRI examination (optional): For patients with metastases shown by baseline cranial MRI/enhanced CT, this item is required at each subsequent efficacy evaluation. For patients with negative baseline brain metastases, this item is optional and is only required when there are symptoms. Whole-body bone scan (optional): It is performed for patients with only symptoms of bone metastasis or those who need to confirm CR. PET-CT is optional. Note: If new lesions are suspected at any site outside the follow-up, imaging examinations should be performed appropriately.
14. From the diagnosis of NSCLC to any time point before the enrollment of this treatment, the gene mutation detection of the primary and metastatic tumor can be performed, including EGFR (exon 18, 19, 20, 21) mutation, ALK and ROS-1 gene rearrangement.
15. Blood sample collection: All the following samples should be clearly marked with the clinical trial code, patient ID number, collection date and time, and sample type.

(1) One EDTA anticoagulation tube and one coagulation promoting tube of 10 ml venous blood should be collected, respectively.

(2) Plasma and PBMC from anticoagulant blood should be separated within 2 hours and stored at minus 80 degrees.

(3) Pro-coagulant blood is separated within 2 hours after collection, and stored at minus 80 degrees

1. Tumor tissue biopsy: all the following samples should be clearly marked with the clinical trial code, patient ID number, collection date and time, and sample type.
2. A 16-18G biopsy gun is used to take at least 5mm*5mm tumor tissues from the target lesions.
3. One piece is placed in formalin and sent to the pathology department for paraffin embedding;
4. The other one is placed in RNA later at 4°C overnight and then stored at minus 80°C for long-term storage.

**5 Treatment**

The combination of SBRT with sintilimab and GM-CSF is applied. One lesion (primary or metastatic lesion) is selected for SBRT, with the dose of 24Gy/3Fx. Within Two weeks after SBRT is completed, Sintilimab and GM-CSF treatment are initiated and used in combination, with the date recorded as d1. GM-CSF is continuously used for 14 days (d1-d14) at the dose of 125 μg/m^2^. Sintilimab is administrated at the dose of 200 mg/time once every 3 weeks from d1 (d21n +1, n≥0), 35 cycles in total (2 years), or until the investigator deems that the patient needs to be discontinued due to treatment-related toxicity or disease progression. The overall response rate and toxicity were assessed periodically.

**5.1 SBRT**

One primary or metastatic lesion with a size of 1-5 cm, a clear boundary, little influence on surrounding normal tissues and no previous irradiation is selected and performed SBRT. The total dose of each lesion is 24 Gy/3 Fx (8 Gy/Fx). Each lesion is irradiated once a day for 3 consecutive days. Taking into account the failure of the radiotherapy machine and other factors, it is allowed to complete 3 fractions within 5 days.

**The principle of lesion selection:**

1. Try to choose lesions with less damage to normal tissues.
2. Try to choose the largest one among all the available lesions, but the size should not exceed 5cm.
3. For bone metastases, if there is no obvious soft tissue mass, they should not be selected for SBRT.
4. Brain metastases should not be selected for SBRT treatment.
5. The lesions that are easier to undergo biopsy should be kept for biopsy, and not be selected for SBRT.

**5.1.1 Positioning and Simulation**

Equipment: Analog CT

Scanning range: The target area and all organs at risk should be covered. The scanning length is extended by at least 5-10cm to the cephalic and crural sides of the boundary of the target area.

Scanning layer thickness: 3 mm/layer.

Contrast agent application: If appropriate.

**5.1.2 Definition of target volume**

**GTV:** The visible tumor displayed during CT simulation. MRI, PET and other information should be referred to for the evaluation of tumor size and extent when necessary.

CTV: In this study, CTV=GTV

ITV: Respiratory motion control technology is required for lesions that are significantly affected by respiratory motion. 4DCT technology is strongly recommended, the respiratory motion control methods of which include chest/abdominal compression, active respiratory control technology (ABC), and respiratory gating technology.

**PTV:** Cone-beam CT (cone-beam CT) must be performed to verify the body position before each SBRT treatment, so the external placement boundary from ITV to PTV is 5mm.

**5.1.3 Radiation dose**

The prescribed dose of PTV is 24 Gy/3Fx. Tumor prescription dose requirements include that the dose is normalized to the average dose point of PTV, that 95% PTV receives the prescribed dose, that 99% PTV receives 95% of the prescribed dose, and that the area < 95% of the prescribed dose cannot fall on the GTV.

**5.1.4 Dose constrains for organ at risk (according to AAPM Task Group 101 document)**

**Table 2. Dose constrains for OAR**

| **Tandem organs** | **Volume** | **Maximum volume dose (Gy)** | **Maximum point dose (Gy)** | **Adverse events (≥ grade 3)** |
| --- | --- | --- | --- | --- |
| Visual pathway | <0.2cc | 15.3Gy | 17.4Gy(5.8Gy/Fx) | Neuritis |
| Cochlea |  |  | 17.1Gy(5.7Gy/Fx) | Hearing loss |
| Brainstem (not spinal cord) | <0.5cc | 18Gy(6Gy/Fx) | 23.1Gy(7.7Gy/Fx) | Cranial neuropathy |
| Vertebral body  spinal cord | <0.35cc  <1.2cc | 18Gy(6Gy/Fx)  12.3Gy(4.1Gy/Fx) | 21.9Gy(7.3Gy/Fx) | Myelitis |
| Spinal cord segmentation (upper and lower 5-6 mm each) | <10% segment volume | 18Gy(6Gy/Fx) | 21.9Gy(7.3Gy/Fx) | Myelitis |
| Horsetail | <5cc | 21.9Gy(7.3Gy/Fx) | 24Gy(8Gy/Fx) | Neuritis |
| Sacral plexus | <5cc | 22.5Gy(7.5Gy/Fx) | 24Gy(8Gy/Fx) | Neuropathy |
| Esophagus | <5cc | 17.7Gy(5.9Gy/Fx) | 25.2Gy(8.4Gy/Fx) | Stenosis/fistula |
| Brachial plexus | <3cc | 20.4Gy(7.5Gy/Fx) | 24Gy(8Gy/Fx) | Neuropathy |
| Heart/pericardium | <15cc | 24Gy(8Gy/Fx) | 30Gy(10Gy/Fx) | Pericarditis |
| Great blood vessels | <10cc | 39Gy(13Gy/Fx) | 45Gy(15Gy/Fx) | Aneurysm |
| Trachea and main bronchus | <4cc | 15Gy(5Gy/Fx) | 30Gy(10Gy/Fx) | Stenosis/fistula |
| Bronchial branches | <0.5cc | 18.9Gy(6.3Gy/Fx) | 23.1Gy(7.7Gy/Fx) | Stenosis with atelectasis |
| Ribs | <1cc  <1cc | 28.8Gy(9.6Gy/Fx)  30.0Gy(10.0Gy/Fx) | 36.9Gy(12.3Gy/Fx) | Pain or fracture |
| Skin | <10cc | 30Gy(10Gy/Fx) | 33Gy(11Gy/Fx) | Ulcer |
| Stomach | <10cc | 16.5Gy(5.5Gy/Fx) | 22.2Gy(7.4Gy/Fx) | Ulcer/fistula |
| Bile duct |  |  | 35.7Gy(11.9Gy/Fx) | narrow |
| Duodenum | <5cc  <10cc | 16.5Gy(5.5Gy/Fx)  11.4Gy(3.8Gy/Fx) | 22.2Gy(7.4Gy/Fx) | ulcer |
| Jejunum/ileum | <5cc | 17.7Gy(5.9Gy/Fx) | 25.2Gy(8.4Gy/Fx) | Inflammation/obstruction |
| Colon | <20cc | 24Gy(8Gy/Fx) | 28.2Gy(9.4Gy/Fx) | Colitis/Fistula |
| Rectum | <20cc | 24Gy(8Gy/Fx) | 28.2Gy(9.4Gy/Fx) | Proctitis/Fistula |
| Ureter |  |  | 48.9Gy(16.3Gy/Fx) | Narrow |
| Bladder wall | <15cc | 16.8Gy(5.6Gy/Fx) | 28.2Gy(9.4Gy/Fx) | Cystitis/Fistula |
| Vaginal sperm | <3cc | 21.9Gy(7.3Gy/Fx) | 42Gy(14Gy/Fx) | Impotence |
| Femoral head (left/right) | <10cc | 21.9Gy(7.3Gy/Fx) |  | Necrosis |
| Renal Hilar/Main Vessel | <2/3 volume | 18.6Gy(6.2Gy/Fx) |  | Malignant hypertension |
| **Parallel organs** | **Critical volume** | **Maximum critical volume dose (Gy)** |  | **Adverse events (≥ grade 3)** |
| Lung (left/right) | 1500cc | 11.6Gy(2.9Gy/Fx) |  | Basic lung function |
| Lung (left/right) | 1000cc | 12.4Gy(3.1Gy/Fx) |  | Pneumonia |
| liver | 700cc | 19.2Gy(4.8Gy/Fx) |  | Basic liver function |
| Renal cortex (left/right) | 200cc | 16.0Gy(4.0y/Fx) |  | Basic kidney function |

**5.1.5 Radiotherapy plan**

The linear accelerator is used, which is 6MV X-rays, and the width of MLC is ≤ 5mm. The radiation field is designed with a fixed field and/or a dynamic rotating field. Pulmonary heterogeneity correction was performed when calculating the dose. The shape of the irradiation field is designed by Beams Eye View (BEV). An isotropic grid system less than or equal to 2 mm is used when calculating the volume. The treatment plan is determined by comprehensive evaluation of Dose Volume Histogram (DVH), isodose line graphs, two-dimensional isodose line and Color washing.

**5.1.6 Implementation and verification of radiotherapy plan**

Cone-beam CT must be performed to verify the body position, and the placement error must be corrected before each SBRT.

**5.2 Sintilimab**

Starting on the second day (d1) after the completion of SBRT treatment, Sintilimab was given intravenously once every 3 weeks at a dose of 200 mg. Blood routine, blood biochemistry, coagulation function, and thyroid function should be checked before each administration, in order to evaluate the related adverse events. During each 3 courses of treatment (every 9 weeks), the efficacy is evaluated (according to the RECIST1.1), and the treatment is continued until disease progression, death, unacceptable toxicity or up to 35 cycles (2 years) or the patient withdraws informed consent. See 5.2.1-5.2.8 for the principles of Sintilimab use, withdrawal, resumption of medication, infusion reaction management, dose adjustment, toxicity management, concomitant treatment and drug management.

**5.2.1 Use of Sintilimab**

The main active ingredient of IBI308 is a recombinant fully humanized anti-programmed death receptor 1 monoclonal antibody with a concentration of 10 mg/mL. This product is a clear, colorless liquid, free of foreign matter, flocculation and precipitation. Excipients include 140 mmol/L mannitol, 25 mmol/L histidine, 20 mmol/L sodium citrate dihydrate, 50 mmol/L sodium chloride, 0.02 mmol/L disodium edetate (ethylenediamine tetra Disodium acetate), 0.2 mg/mL polysorbate 80, pH 6.0.

The smallest packaging unit of IBI308 is a box, and each box contains 1 Sintilimab injection packaged in a vial. The packaging box is printed with the name of the drug, dosage form, specification, drug code, production batch number, expiration date, storage conditions and information of the sponsor. The same information is printed on the label of the vial and the box, but there is no information on the dosage form, precautions, usage and dosage on the label of the vial. The labels of all packaging boxes and vials are marked "For clinical research use only". Sintilimab products are stored in the dark at 2~8℃, and the validity period is 24 months. If there are quality problems such as turbidity and precipitation in the injection, it should be sealed up immediately, and Innovent Biologics (Suzhou) Co., Ltd. (hereinafter referred to as "Innovent Biologics") should be notified immediately.

The preparation and infusion process of IBI308 is as follows:

1. Draw out 2 bottles of IBI308 injection completely, add 100mL 0.9% (weight/volume) sodium chloride sterile saline intravenous infusion bag, and record the start time of preparation.

2. Gently invert the infusion bag to mix to ensure the uniformity of the medicine in the infusion bag and avoid foaming due to violent shaking. If a large amount of foam is generated, the drug should be allowed to stand until the foam disappears.

3. The drug is administered through a filter equipped with 0.2~1.2μm (it is recommended that the infusion time be controlled within 30-60 minutes), and the start time and end time of the drug delivery are recorded.

Note: Before configuration, confirm that the IBI308 injection is transparent, and there are no quality problems such as turbidity or precipitation; ensure that the time from the first bottle of IBI308 injection to the end of administration does not exceed 24 hours (the storage condition of the prepared drug is kept in a refrigerator refrigerated at 2~8℃); avoid mixing other drugs; avoid intravenous bolus injection.

**5.2.2 Principles of Suspension and Permanent Discontinuation of Sintilimab**

The dose of Sintilimab is not allowed to be adjusted during the entire study. The principles of suspension and permanent discontinuation of Sintilimab are shown in the table below.

| **Adverse events related to Sintilimab** | **Severity** | **Dose adjustment** |
| --- | --- | --- |
| Pneumonitis | Grade 2 | Suspend dosing ^a^ |
|  | Recurrent grade 2 or grade 3-4 | Permanent discontinuation |
| Diarrhea/Enterocolitis | Grade 2 or 3 | Suspend dosing ^a^ |
|  | Grade 4 | Permanent discontinuation |
| Dermatitis | Grade 3 | Suspend dosing ^a^ |
|  | Grade 4 | Permanent discontinuation |
| Hepatitis | For subjects with normal baseline ALT, AST, or TBIL, Grade 2 elevation in AST, ALT, or TBIL;  For subjects with baseline AST, ALT, or TBIL>ULN, elevation in AST, ALT or TBIL ≥50% and duration<7 days | Suspend dosing ^a^ |
|  | For subjects with normal baseline ALT, AST, or TBIL, Grade3-4 elevation in AST, ALT, or TBIL; For subjects with baseline AST, ALT, or TBIL>ULN, AST, ALT, or TBIL increased by ≥50% and the duration ≥ 7 days | Permanent discontinuation |
| Hypophysitis | Grade 2 | Withhold dosing ^b^ |
|  | Grade 3 or 4 | Permanent discontinuation |
| Adrenal insufficiency | Grade 2 | Suspend dosing ^b^ |
|  | Grade 3 or 4 | Permanent discontinuation |
| Hyperthyroidism | Grade 3 or 4 | Permanent discontinuation |
| Type I diabetes | Grade 3 | Suspend dosing ^b^ |
|  | Grade 4 | Permanent discontinuation |
| Renal insufficiency | Grade 2 or 3 | Suspend dosing ^b^ |
|  | Grade 4 | Permanent discontinuation |
| Neurotoxicity | Grade 2 | Suspend dosing ^a^ |
| Neurotoxicity | Grade 3 or 4 | Permanent discontinuation |
| Other AE | First appearance Grade 3 | Suspend dosing ^a^ |
|  | Second occurrence Grade 3 | Permanent discontinuation |
|  | Cannot drop to grade 0-2/baseline level within 7 days or return to grade 0-1 within 14 days/Baseline level 3 AE | Permanent discontinuation |
|  | Grade 4 AE | Permanent withdrawal ^c^ |

**Table 3. Suspension and permanent withdrawal of Sintilimab**

a: Resume administration after the symptoms improved to grade 0-1 or baseline level.

b: For hypophysitis, adrenal insufficiency, thyroid insufficiency/hypothyroidism, and type 1 diabetes, Sintilimab can be restarted if they are fully controlled and require only physiological hormone replacement therapy.

c: For abnormal grade 4 laboratory test results, the decision to terminate the medication should be based on the accompanying clinical symptoms/signs and the clinical judgment of the investigator.

The maximum interval allowed for drug suspension is 12 weeks. If it is not possible to return to the state where Sintilimab can be re-used within 12 weeks, the subject will permanently stop Sintilimab and enter the follow-up phase. Except for the following two cases:

1. Due to the application of glucocorticoids in the treatment of irAE, the process of glucocorticoid reduction led to the suspension of Sintilimab for more than 12 weeks. In this case, it is necessary to discuss with the sponsor's medical manager to decide whether to continue Sintilimab treatment. The imaging examination to evaluate the efficacy is carried out as planned, and is not affected by the suspension of medication.

2. Sintilimab was suspended for more than 12 weeks due to treatment related AEs unrelated to Sintilimab. In this case, it is necessary to discuss with the sponsor to decide whether to continue Sintilimab treatment. The imaging examination to evaluate the efficacy is carried out as planned, and is not affected by the suspension of medication.

**5.2.3 Restoration of Sintilimab**

Resuming use of Sintilimab requires that treatment related AEs to return to grade 0-1 or baseline, with an ECOG PS score of 0-1.

**5.2.4 Management of Sintilimab-related infusion reactions**

Sintilimab may cause severe or life-threatening infusion reactions, including severe hypersensitivity or allergic reactions. Signs and symptoms usually appear during the drug infusion or shortly after the infusion, and can usually be managed after the infusion is completed. It can be completely resolved within 24 hours. Guidelines for the management of sintilimab-related infusion reactions are shown in the table below.

| **NCI CTCAE classification** | **Treatment** | **Pretreatment medication during subsequent administration** |
| --- | --- | --- |
| Level 1  Mild reaction; no need to interrupt the infusion; no intervention | According to the patient's medical indications, strengthen the monitoring of their vital signs until the researcher believes that the subject's condition is stable. | no |
| Level 2  Need treatment or interruption of the infusion, symptomatic treatment (such as antihistamines, non-steroidal anti-inflammatory drugs [NSAIDS], anesthetics, intravenous fluids) should be given as soon as possible and a rapid response; preventive medication for ≤24 hours should be taken | Stop the infusion and monitor the symptoms. Other appropriate medications may include but are not limited to: intravenous infusion of antihistamines, NSAIDS, Paracetamol anesthetic  According to the patient's medical indications, strengthen the monitoring of their vital signs until the researcher believes that the subject's condition is stable.  If the symptoms are relieved within one hour after stopping the drug infusion, the infusion can be restarted at 50% of the original infusion rate (for example, reduced from 100 mL/h to 50 mL/h). Otherwise, the medication should be suspended until the symptoms are relieved, and the subject should receive pretreatment medication before the next scheduled dosing.  For subjects who still have Grade 2 toxicity even after receiving adequate pretreatment medication, further study drug treatment should be permanently discontinued. | The following pretreatment medications are acceptable for 1.5 hours (±30 minutes) before Sintilimab:  Oral 50 mg diphenhydramine (or an equivalent dose of antihistamine).  500-1000 mg acetaminophen (or an equivalent dose of antipyretic) is taken orally. |
| Level 3 or 4  Level 3:  Long duration (that is, failure to respond quickly after symptomatic medication and/or short-term interruption of infusion); symptoms relapse after initial improvement; hospitalization is required due to other clinical sequelae (such as kidney damage, lung infiltration)  Level 4:  Life-threatening; requires pressor agents or ventilatory support | Stop the infusion. Other appropriate medications may include but are not limited to: epinephrine**, intravenous infusion of antihistamines, NSAIDS, Paracetamol anesthetic, Oxygen booster drugs, Corticosteroids.  Should strengthen the monitoring of the patient’s vital signs based on the patient’s medical indications until the investigator believes that the subject’s condition is stable.  May require hospitalization.  **If an allergic reaction occurs, epinephrine should be used immediately.  Subjects should permanently stop further study drug treatment. | No follow-up administration |
| Appropriate first aid equipment should be provided in the ward, and the physician should be contacted at any time during the administration period. For further information, please refer to the Common Terminology Standard for Adverse Events (CTCAE) Version 5.0[(Http://ctep.cancer.gov)](http://ctep.cancer.gov/) | | |

**5.2.5 Other permitted dose adjustments of Sintilimab**

In conditions other than treatment-related AEs, such as medical/surgical events or management reasons not related to the study treatment, sintilimab treatment may be discontinued. Subjects should restart the study treatment within 3 weeks after the planned discontinuation of medication, unless there are other discussions with the sponsor. The reason for discontinuing medication should be recorded in the subject’s study record.

**5.2.6 Principles for the treatment of the toxicity of immune-checkpoint inhibitors**

AEs related to sintilimab exposure may be caused by immunological mechanisms. These immune-related AEs (irAE) may occur within a short period of time after the first administration or several months after the last administration of Sintilimab, and may affect more than one body systems at the same time. Therefore, early detection and initiation of treatment is essential to reduce complications. Based on current clinical trial data, most irAEs are reversible and can be managed by interruption of sintilimab, administration of glucocorticoids, and/or other supportive therapies. For suspected irAEs, make sure to perform an appropriate evaluation to confirm the cause or rule out other causes. Other procedures or tests such as bronchoscopy, endoscopy or skin biopsy may be included as part of this assessment. Based on the severity of the irAE, suspend or permanently stop sintilimab treatment and administration of glucocorticoids. The dose adjustment and toxicity management guidelines for potential irAEs can be found in the "Immune-related Adverse Events Management Manual" provided by the sponsor.

**5.2.7 Concomitant therapy**

5.2.7.1 Permitted concomitant treatment

Medications determined by the investigator to meet the requirements of the protocol (for example, for the treatment of disease-related symptoms and concomitant treatment of various AEs related to the treatment). Subjects who need long-term medication due to underlying diseases such as hypertension and diabetes can continue their medication. Local glucocorticoid medications are allowed, such as topical skin application, eye drops, nasal spray, and inhalation. Subjects who routinely take non-steroidal anti-inflammatory drugs (NSAIDs) or salicylic acid drugs (equivalent to 1.3g aspirin per day) should stop the above drugs. Subjects taking long half-life NSAIDs or salicylic acid drugs (such as naproxen, piroxicam, diflunisal, or nalbumetone) need to stop the drugs 5 days before to 2 days after Sintilimab initiation.

5.5.2 Prohibited concomitant treatment

Subjects are prohibited from receiving the following treatments during the treatment period of this study: Biological therapy with anti-tumor effect (except for cytokine drugs used to treat adverse events caused by chemotherapeutic drugs), and proprietary Chinese medicines with anti-tumor effect; Drugs with immunomodulatory effects, including but not limited to non-specific immunomodulators (such as thymosin, interferon, interleukin, immunoglobulin, gamma globulin) and Chinese patent medicines with immunomodulatory effects, etc. Chemotherapy not specified in this regimen. Live vaccination within 30 days before the first administration of Sintilimab and during the study. Live vaccines include, but are not limited to, the following: measles, mumps, rubella, varicella, yellow fever, rabies, BCG, typhoid (oral) vaccine. It is allowed to receive inactivated virus vaccines against seasonal influenza, but it is not allowed to receive live attenuated influenza vaccines for intranasal inoculation. Corticosteroids. Allows the use of inhaled steroids as part of a fixed treatment for asthma or chronic obstructive pulmonary disease (COPD). Allows corticosteroids to be used in the treatment of adverse events with underlying immune causes. After consultation with the sponsor, the use of physiological doses of corticosteroids can be approved. Note: Prophylactic corticosteroids are allowed to avoid allergic reactions (for example, pretreatment prior to administration of intravenous contrast agents or chemotherapy drugs). According to the investigator's assessment, subjects who need to use any of the above treatment methods for clinical treatment should be excluded from the trial. Subjects can receive other medications deemed medically necessary by the investigator. It is important for researchers to review each drug (prescription and over-the-counter) that subjects received before the start of the study and during each study visit. At each visit, the subject must be asked about any new drugs received. In order to reduce the risk of adverse drug interactions, all measures must be taken to limit the number of concomitant medications that are really necessary. During the administration period, you should avoid receiving hepatotoxic drugs (that is, drugs that are warned of hepatotoxicity in the product instructions). Researchers are encouraged to [review each](http://www.livertox.nih.gov/) potential hepatotoxic drug by searching the www.livertox.nih.gov website.

**5.2.8 Drug management**

5.2.8.1 Drug management

The investigational drug in this study is Sintilimab (IBI308). Sintilimab should be refrigerated at 2～8℃, protected from light, and stored in a dry place. Do not freeze. All test drugs are transported to each research center in the cold chain, and should be kept and distributed by special personnel. Test drugs should be stored in a refrigerator that only authorized personnel can open. After receiving the drugs, the investigator should confirm that the drug transportation temperature is within the specified range, sign for receipt after verification, and store at the specified temperature. If the temperature is abnormal during transportation or storage in the research center, the drug should be transferred to the specified temperature as soon as possible, and the subject should not be used for the time being, and reported to in time, and deal with it according to Innovent Biologics’ opinion. All experimental drugs provided by the sponsor can only be used in this experimental study, and shall not be used for purposes other than those specified in this protocol. The investigator must promise not to provide the trial drug to anyone unrelated to the trial. Sintilimab that has been discontinued due to special reasons should be stored under the same storage conditions until the inspector has completed the inventory and check, and arrange for recycling.

5.2.8.2 Drug recovery and destruction

In this study, the used research drug container can be destroyed on the spot according to the applicable guidelines and operating procedures established by the research center and local institutions. All unused research drugs shall be recycled to Innovent Biologics for unified destruction after the research is completed/terminated or after the expiry date.

5.2.8.3 Records of study drugs

The designated personnel of the research center shall make timely records of the receipt, distribution, use, inventory, destruction, recovery, and destruction of research drugs in accordance with the requirements of relevant regulations and guidelines.

**5. 3 GM-CSF**

Commodity name: Terli; Manufacturer: Xiamen Tebao Biological Engineering Co., Ltd.

It starts on the second day after the completion of SBRT treatment (the first day of Sintilimab), and is applied for a total of 14 days. The dose is 125μg/m2. During the period (d1-14), a total of 3 peripheral blood routine checks (d5/9/14) are required. The adverse events and treatment principles are shown in the table below.

| **AEs related to GM-CSF** | **Symptoms** | **Discontinuation and treatment** |
| --- | --- | --- |
| Cold-like symptoms | Fever, fatigue, myalgia, etc. | No need to stop the drug, NSAIDS can be used for symptomatic treatment or preventive use |
| Bone pain | Grade 2 | Suspend dosing and resume dosing after symptoms disappear |
|  | Grade 3-4 | Permanent discontinuation |
| Diarrhea/abdominal pain | Grade 2 | Suspend the administration, symptomatic treatment, resume administration after the symptoms disappear |
|  | Grade 3-4 | Permanent discontinuation |
| Dermatitis | Grade 2 | Suspend the administration, treatment with corticosteroids, and resume administration after the symptoms disappear |
|  | Grade 3-4 | Permanent discontinuation |
| Elevated white blood cells | WBC≥40*10^9/L | Suspend the administration, no therapy, resume the administration after WBC≤10*10^9/L |
|  | WBC≥100*10^9/L | Suspend the administration, orally take hydroxyurea daily 20-30mg/kg or leukocyte apheresis, and resume administration after leukocyte ≤10*10^9/L |
| First-dose reaction | Transient hypoxia  or hypotension | Suspend dosing and resume dosing after symptoms disappear |
| Respiratory distress syndrome | Acute dyspnea, refractory hypoxemia, diffuse infiltrating shadows of both lungs on chest X-ray | Permanently discontinue the drug  give oxygen and glucocorticoid therapy |
| Other conditions that the investigator believes need to be stopped |  | Suspend or permanently discontinue the drug |

**6 Statistical analysis**

**6.1 Calculation of sample size**

This study uses the objective response rate (ORR) as the primary end point. This single-arm study requires 56 eligible patients. Considering a 10% dropout rate, a total of 63 patients will be enrolled.

The research hypothesis is based on Checkmate 017 and Checkmate 057 study, which reported an ORR of 20%. If the ORR of the experimental treatment in this study is 20%, it is not worthy of further study. But if the ORR of the experimental treatment in this study is 38%, the study is considered successful. The purpose of this study is to distinguish ORR between 20% and 38%. The statistical assumptions are: the null hypothesis ORR<=20%, and the opposite hypothesis ORR>=38%. If 17 or more of the 56 eligible patients are CR or PR, this study is deemed successful and is worthy of further study. With a one-sided type one error rate (alpha) equal to 0.0432, this study has at least 90% statistical power to reject the null hypothesis.

**6.2 Safety Run-in**

In this study, dose-limiting toxicity (DLT) includes the following three conditions and asymptomatic biochemical abnormalities without clinical significance are not included:

(1) Grade 3 toxicity lasts for more than 7 consecutive days;

(2) Grade 4 toxicity

(3) Grade 5 toxicity that could not be ruled out the association with the trial treatment

To prevent the experimental treatment from causing excessive toxicity to patients, we will monitor the toxicity of the first 20 patients receiving the experimental treatment. Within 30 days after the first dose of sintilimab and GM-CSF, if 7 or more of these 20 patients develop any of the pre-defined DLTs, enrollment will be suspended immediately. After reviewing the data and other details, the research team will decide whether to revise the trial's treatment plan to reduce the incidence of DLT or to terminate the entire trial. This safety run-in phase of the trial has the following statistical characteristics. In this treatment scenario, it is assumed that a DLT rate of 25% or lower is acceptable, while a DLT rate of 45% or higher is not acceptable. When the DLT rate is equal to 25%, the probability of termination of the test in the Run-in phase is 0.1018; when the DLT rate is equal to 30%, the probability of termination of the test in the Run-in phase is 0.7480. In addition, if there are more than 4 deaths caused by treatment in the first 20 treated patients within the first 60 days of treatment, the trial will be stopped immediately and investigation will be carried out.

**6.3 Patient recruitment**

Assuming that the monthly recruitment rate is 4-5 patients, this trial is expected to complete the recruitment of patients 13-16 months after the start of the study. The last patient was followed up for another 25 months after enrollment.

**6.4 Baseline characteristic evaluation**

Baseline data include: demographic characteristics, tumor characteristics, medical history, previous anti-cancer treatments, combined medications, vital signs, etc. For measurement data, the mean, standard deviation, median, minimum, and maximum are used for description; for count data, frequency and percentage are used for description.

**6.5 Study Endpoint**

**6.5.1 Primary endpoint**

**Overall response rate (ORR):** The ratio of the total number of patients achieving CR or PR to the total number of evaluable cases according to the RECIST1.1. The data will be expressed as a two-sided confidence interval.

**6.5.2 Secondary endpoint**

**Treatment-related adverse events (TRAE):** Treatment-related toxic reactions according to the CTCAE 5.0.

**Out-of-field (abscopal) response rate (ASR):** Out-of-field response was defined as reduced by ≥30% of the longest diameter from baseline in any un-irradiated RECIST1.1 evaluable target lesions. ASR was defined as the percentage of patients achieving out-of-field response.

**Overall survival (OS):** The time interval from the first day of enrollment to death from any cause. Patients who are still alive at the time of analysis will be censored by the date of last contact until cut-off date. The Kaplan-Meier method will be used to estimate the survival curves of the two groups, and the median OS (90% two-sided confidence interval) will be calculated.

**Progression free survival (PFS):** The time interval from enrollment to the first documentation of disease progression or death due to any cause. Patients who are still alive without disease progression until data cut-off will be censored by the time of the most recent radiographic follow-up. The Kaplan-Meier method was used to estimate the survival curves of the two groups, and the median PFS (90% two-sided confidence interval) was calculated.

**6.6 Safety analysis**

Adverse events and their most serious reaction levels will be summarized in accordance with the standards of NCI CTCAE Version 5.0. Adverse events will also be summarized according to the severity of the event and its relationship with the study drug. The descriptive summary of laboratory test values ​​is mainly for outliers. Laboratory abnormalities will also be summarized according to the most severe level in NCI CTCAE Version 5.0.

**6.7 Plan of Statistical Analysis**

We will calculate the ORR and the 95% confidence interval. According to the type and severity of adverse events, we will list the number and rate of therapeutic adverse events. We use the Kaplan-Meier survival curve to describe the overall survival time (OS) and progression-free time (PFS) of the patients.

**7 Adverse event report and management**

**7.1 Definition of adverse events**

An adverse event (AE) is defined as any adverse medical event that occurs in the clinical trial subject from the signing of the informed consent form until 90 days after the last use of the study drug, regardless of whether there is a causal relationship with the study drug, it is judged as an adverse event. AE includes but is not limited to the following situations:

- Exacerbation of the original (before entering the clinical trial) medical condition/disease (including symptoms, signs, and exacerbations of abnormal laboratory tests);
- Any new adverse medical conditions (including symptoms, signs, newly diagnosed diseases);
- Abnormal laboratory test values ​​or results with clinical significance.

**7.2 Definition of serious adverse events**

Serious adverse events are those that meet at least one of the following criteria:

Causes death, except for deaths caused by disease progression of the research indication.

Life-threatening ("life-threatening" in the definition is an AE that is at risk of death when the subject occurs, and does not include AEs that may cause death if the event worsens).

Need to be hospitalized or extend the length of hospitalization, excluding the following situations:

-  Rehabilitation institutions
-  Nursing home
-  Admission to the routine emergency room
-  one-day surgery (such as outpatient/same-day/ambulatory surgery)
-  Hospitalization or prolongation of hospital stay unrelated to worsening AE is not SAE in itself. E.g: Hospital admission due to pre-existing diseases, no new adverse events, no exacerbations of pre-existing diseases (such as: to check the laboratory abnormalities that have persisted before the test); hospitalization for management reasons (such as: annual physical examination ); hospitalization specified in the trial protocol during the clinical trial (such as operation as required by the trial protocol); elective hospitalization that has nothing to do with worsening adverse events (such as elective surgery); scheduled treatment or surgery should be included in the entire trial protocol and / Or recorded in the subject's personal baseline data; admitted to the hospital only for the use of blood products.

 Cause permanent or severe disability/incapacity.

 Cause congenital abnormalities/birth defects.

Other important medical events: defined as events that harm subjects or require medical intervention to prevent any of the above from happening.

**7.3 Assessment of adverse events**

The investigator will evaluate all adverse events in accordance with NCI Common Adverse Events (CTCAE) version 5.0. Any adverse event that changes the CTCAE level will be recorded in the adverse event case report form/worksheet. All adverse events, regardless of the CTCAE grade, must be evaluated whether they are serious adverse events. For specific details, please refer to the table below.

Adverse event assessment rules

| **CTCAE**  **Grade** | **Level 1** | **Mild; asymptomatic or mild; only clinical or diagnostic manifestations; no intervention required** |
| --- | --- | --- |
|  | **Level 2** | **Moderate; requires minimal, partial or non-invasive intervention; restricts daily activities of the appropriate age** |
|  | **Level 3** | **Severe or important medical events require medication, but they are not immediately life-threatening; hospitalization or extended hospitalization; disability; limitation of self-care ability ADL** |
|  | **Level 4** | **Life-threatening consequences; urgent intervention required** |
|  | **Level 5** | **AE-related deaths** |
| **Severity** | A serious adverse event is any of the following adverse events that occur at any dose or during any use of the study drug: | |
|  | †Causes **death;** | |
|  | †Life **threatening; or** in the eyes of the researcher, the occurrence of the event puts the subject at immediate risk of death (Note: This does not include adverse events that may lead to death if they occur in a more serious form); | |
|  | †Causes **permanent or severe disability/dysfunction; (seriously interferes with the** ability to carry on a normal life); | |
|  | †Causes **or prolongs existing hospitalization; (Hospitalization** is defined as a hospitalization, regardless of the length of hospitalization, even if the hospitalization is only a preventive measure for continued observation. Note: Hospitalization [including hospitalization due to elective surgery] due to an existing disease that did not worsen during the study period does not constitute a serious adverse event. Pre-existing diseases refer to clinical diseases that were diagnosed before the use of study drugs and recorded in the patient's medical history); | |
|  | †Congenital **abnormalities/birth defects; (the offspring of** subjects who use the product, regardless of the time of diagnosis); | |
|  | **Other important medical events;** although it will not lead to death, life-threatening, or hospitalization, based on appropriate medical judgment, the event may endanger the subject and may require medical or surgical intervention to prevent the previously listed ( One of the outcomes marked as †) above, such an event can also be regarded as a serious adverse event. | |
| **duration** | Record the start and end date of the adverse event. If it is less than 1 day, indicate the appropriate length of time and unit. | |
| **Take measures** | Did the adverse event lead to the discontinuation of the study drug? | |
| **Relationship with study drug** | Did the study drug cause the adverse event? A medically qualified investigator needs to provide a causal evaluation result between the study drug and the adverse event. The investigator signs the original document or worksheet with the date (initial acronym) to support the causality evaluation on the AE form to ensure that a medically qualified causality evaluation is performed. This signed document must be kept within the required regulatory time frame. The purpose of the following standards is to serve as a reference guide to assist investigators in assessing the relationship between trial drugs and adverse events based on existing information.  **The following elements are used to evaluate the relationship between study drugs** and AEs; the greater the correlation between the items and their corresponding elements (in terms of quantity and/or intensity), the greater the likelihood that the study drug will cause adverse events; | |
|  | **Exposure** | Whether there is evidence that the subject is actually exposed to the test drug, such as: a true and credible past medical history, acceptable compliance assessment (drug count, log, etc.), expected pharmacological effects, drug/metabolite measurement in samples collected in vivo ? |
|  | **Time** | Is there a reasonable time sequence between adverse events and trial drug treatment?  Does the timing of the adverse event coincide with the drug-induced adverse event? |
|  | **Possible reason** | Whether the adverse event cannot be explained by other causes, such as underlying diseases, other drugs/vaccines, or other host or environmental factors |
|  | **Deprovocation test** | Is the study drug discontinued or reducing the dose/exposure/frequency?  If yes, can the AE be cured or improved?  If it is, it means that the deprovocation test is positive. If not, it means that the deprovocation test is negative.  Note: This standard does not apply if the following conditions occur: (1) An adverse event causes death or permanent disability; (2) The AE is cured/improved despite the continued use of the study drug; (3) The trial is a single dose trial; (4) The study drug has been used only once. |
|  | **Re-provocation test** | Is the subject repeatedly exposed to the study drug in this trial?  If yes, is the AE recurring or worsening?  If it is, then the provocation test is positive. If not, then the provocation test is negative.  Note: This criterion does not apply if the following conditions occur: (1) the initial AE resulted in death or permanent disability, or (2) the trial was a single-dose trial, or (3) the study drug was used only once.  Note: If a re-provocation test is planned for serious adverse events that may be caused by the test drug, or if the test drug is re-exposed, it may cause serious potential risks to the subject/patient. In this case, a re-provocation test is not recommended Unless it is considered that the continued medication may be beneficial to the patient and there is no alternative treatment available, it can only be carried out after obtaining the approval of the sponsor in advance. |
|  | **Consistency with experimental treatment characteristics** | Are the clinical/pathological manifestations of the adverse event consistent with the previous treatment data on the investigational drug or the pharmacology and toxicology trials of such drugs? |
| A medically qualified researcher will report the evaluation results of the relationship in the case report form/worksheet based on his/her best clinical judgment, including considering the relationship between the above factors. | | |
| **Record causality** | | **The following table can be used for causal association assessment (not all criteria need to be met)** |
| **related** | | There is evidence of exposure to study drugs. The time sequence of AE occurrence and study drug administration is reasonable. Compared with other causes, AEs are more likely to be explained by the study drug than other causes. |
| **Irrelevant** | | The subject did not use the study drug or the time relationship between the administration of the study drug and the occurrence of the AE is unreasonable or there are other reasons that are more likely to explain the adverse event, rather than the study drug (also applicable to overdose but no related adverse events Subject). |

**7.4 Record of adverse events**

Researchers should use medical terms/concepts to record AE or SAE. Spoken language and acronyms should be avoided. All AEs (including SAE) should be recorded on the eCRF adverse event form.

7.4.1 Adverse event collection

Researchers learn about adverse events by asking subjects non-inducing questions.

Collect all adverse events, including serious adverse events, whether they are observed by the investigator or spontaneously reported by the subject, from the signing of the informed consent form to 90 days after the last administration, after which, the investigator should report serious adverse events that are considered to be related to the study drug or procedure.

7.4.2 Follow-up of adverse events

Adverse events should be followed up until they return to baseline or Grade 0-1 or the investigator believes that no follow-up is necessary due to reasonable reasons (such as no possibility of recovery or already recovered). If the adverse event cannot be recovered, a reasonable explanation shall be recorded in the eCRF. Regardless of whether it is related to the study drug, the subject’s recovery from AE or SAE and its date should be recorded in the eCRF and medical records.

7.4.3 Content of AE record

The investigator must fully record any adverse event, including diagnosis (if there is no diagnosis, record symptoms and signs including laboratory abnormalities), start and end dates and times (if applicable), CTCAE severity level and changes (events of level 3 or above) , Whether it is a serious adverse event, the measures taken for the study drug, the treatment given due to AE and the result of the event, and the relationship between the adverse event and the study drug.

For serious adverse events, the investigator should also provide the date when the AE meets the SAE criteria, the date the investigator learned of the SAE, the basis for the AE to be an SAE, the date of hospitalization, the date of discharge, the possible cause of death, the date of death, whether an autopsy was performed, and Causality evaluation of the research process, causality evaluation with other drugs, and other possible causes of SAE. The researcher should also provide the basis for the judgment of the relevance and the description of the SAE. In the description of SAE, it is also necessary to include the number, age, sex, height, and weight of the subject; the indications and disease stages of the subject’s trial drug treatment and related systemic conditions; the occurrence, development, outcome and results of the SAE, etc. Clinical course; SAE-related laboratory test results (examination time, unit and normal range must be provided); SAE-related past history, comorbid diseases and their occurrence and duration, etc.; SAE-related medication history, combined drugs and their treatment start, duration, usage and dosage, etc.; detailed information on the start, duration, usage and dosage of study drug treatment.

The matters related to AE records are described as follows:

**Diagnosis, symptoms and signs**

If there is a diagnosis, the diagnosis should be recorded on the eCRF instead of individual symptoms and signs (for example, liver failure, not jaundice, elevated transaminase, and flapping tremor). If the symptoms and signs cannot be determined to be caused by the diagnosis at the time of reporting, they shall be recorded as a separate AE/SAE. If it is determined that the symptoms and signs are caused by the diagnosis, only the diagnosis is reported separately, and the symptoms and signs are included in the diagnosis. AE needs to delete the records of symptoms and signs, and SAE needs to send follow-up update reports.

**Adverse events secondary to other events**

Generally, adverse events secondary to other events (such as caused by other events or clinical sequelae) should be recorded as the primary event, unless the secondary event is serious or is a serious adverse event. However, a secondary event with significant clinical significance should be recorded as an independent adverse event in the eCRF if it occurs at a different time from the primary event. If the relationship between events is not clear, they should be recorded separately in the eCRF.

**Persistent or recurring adverse events**

Persistent adverse events refer to adverse events that have not been relieved between the two evaluation time points of the subject but have persisted. Such adverse events should only be recorded once in the eCRF. The initial severity of the event should be recorded and updated if the event worsens in order to record the most serious degree of the event.

A recurring adverse event refers to an adverse event that has been resolved between two evaluation time points, but later re-occurred. The re-occurrence of adverse events should be recorded separately in the eCRF.

**Abnormal laboratory tests**

Abnormal laboratory test results with clinical significance should be reported as AE. The investigator is responsible for reviewing all laboratory abnormal results and making medical judgments whether each laboratory abnormality should be reported as an AE.

**Death**

During the entire trial period, including all deaths that occurred during the 90-day follow-up period after the last administration, whether or not related to the study drug, should be recorded in the eCRF death report form and reported to the sponsor in a timely manner.

When recording a death event, if the cause of death is clear, the cause of death shall be recorded as an adverse event. The result of the adverse event is death, and the event shall be reported as an SAE; if the cause of death is unknown at the time of reporting, it should be reported in eCRF. The event form was recorded as "death of unknown cause", and the "death of unknown cause" was first reported as SAE, and then the exact cause of death was further investigated.

**Past medical status**

Subjects' existing symptoms/signs during the screening period of the trial should be recorded and reported as adverse events only when the severity, frequency, and nature of the aggravation (except for the deterioration of the disease under study) occur after entering the trial. The record should reflect changes relative to the previous state, such as "increased headache frequency".

**Disease progression**

Disease progression is defined as the deterioration of the subject’s condition caused by the primary tumor targeted by the experimental drug, the appearance of new lesions relative to the primary tumor, or the progression of the original lesions are considered to be disease progression. The expected disease progression is not reported as an AE. Death, life-threatening condition, hospitalization or prolonged hospitalization, permanent or severe disability/incapacity, congenital abnormalities/birth defects and other important medical events, caused by the symptoms and signs of the expected disease progression, are not reported as SAE which needs accelerated reporting.

**New anti-tumor treatment**

Within 90 days of the last administration, if the subject starts a new anti-tumor treatment, only serious adverse events related to the study drug will be recorded and reported.

**7.5 Quick report of SAE and pregnancy**

**SAE report:**

The reporting period of SAE is from signing the informed consent to the serious adverse event occurring within 90 days (including 90 days) after the last administration. If an SAE occurs, the investigator must complete the serious adverse event report form within 24 hours of being notified, and Report to the sponsor, ethics committee and national regulatory authorities. For subjects who have used Sintilimab, in addition to reporting to the above-mentioned departments, the investigator [must also report to Innovent Bio via](mailto:Also%20need%20to%20email%20drugsafety@innoventbio.com%20within%2024%20hours) email drugsafety@innoventbio.com within 24 hours.

If serious adverse events occurring outside the above-mentioned period are deemed to be study drug-related, they should also be reported.

**Pregnancy**

Similar drugs have the safety risk of embryo toxicity, and all subjects who participate in clinical trials with fertility must take effective contraceptive measures.

During the clinical trial, when a female subject who is exposed to the drug becomes pregnant, the subject will leave the trial and report it to the sponsor and Innovent within 24 hours of the researcher's knowledge of the pregnancy.

During the clinical trial, when the partner of the male subject becomes pregnant when the drug is exposed, the subject will continue the clinical trial, and report to the sponsor and Innovent within 24 hours of the researcher being notified of the pregnancy.

The investigator shall continue to monitor the pregnant subjects and follow up the pregnancy results, follow up to 8 weeks after the delivery, and report the results to the sponsor and Innovent.

If the pregnancy result is stillbirth, spontaneous abortion, fetal malformation (any congenital anomaly/birth defect), medical abortion is considered as SAE and needs to be reported in accordance with the SAE process and time limit.

If the subject has an SAE during pregnancy, report it in accordance with the SAE reporting procedure.

**8 Reference**

[ 1 ] Ferlay J，Soerjomataram I，Dikshit R， et al. Cancer incidence and mortality worldwide: sources, methods and major patterns in GLOBOCAN 2012[J]. Int J Cancer，2015，136 (5): E359-386. doi: 10.1002/ijc.29210.

[ 2 ] Jemal A，Siegel R，Xu J， et al. Cancer statistics, 2010[J]. CA Cancer J Clin，2010，60 (5): 277-300. doi: 10.3322/caac.20073.

[ 3 ] Chen W，Zheng R，Zeng H， et al. Annual report on status of cancer in China, 2011[J]. Chin J Cancer Res，2015，27 (1): 2-12. doi: 10.3978/j.issn.1000-9604.2015.01.06.

[ 4 ] Besse B，Adjei A，Baas P， et al. 2nd ESMO Consensus Conference on Lung Cancer: non-small-cell lung cancer first-line/second and further lines of treatment in advanced disease[J]. Ann Oncol，2014，25 (8): 1475-1484. doi: 10.1093/annonc/mdu123.

[ 5 ] Ettinger D S，Aisner D L，Wood D E， et al. NCCN Guidelines Insights: Non-Small Cell Lung Cancer, Version 5.2018[J]. J Natl Compr Canc Netw，2018，16 (7): 807-821. doi: 10.6004/jnccn.2018.0062.

[ 6 ] Gao G，Ren S，Li A， et al. Epidermal growth factor receptor-tyrosine kinase inhibitor therapy is effective as first-line treatment of advanced non-small-cell lung cancer with mutated EGFR: A meta-analysis from six phase III randomized controlled trials[J]. Int J Cancer，2012，131 (5): E822-829. doi: 10.1002/ijc.27396.

[ 7 ] Paz-Ares L，de Marinis F，Dediu M， et al. Maintenance therapy with pemetrexed plus best supportive care versus placebo plus best supportive care after induction therapy with pemetrexed plus cisplatin for advanced non-squamous non-small-cell lung cancer (PARAMOUNT): a double-blind, phase 3, randomised controlled trial[J]. Lancet Oncol，2012，13 (3): 247-255. doi: 10.1016/s1470-2045(12)70063-3.

[ 8 ] Mole R H. Whole body irradiation; radiobiology or medicine?[J]. Br J Radiol，1953，26 (305): 234-241. doi: 10.1259/0007-1285-26-305-234.

[ 9 ] Dudek A M，Garg A D，Krysko D V， et al. Inducers of immunogenic cancer cell death[J]. Cytokine Growth Factor Rev，2013，24 (4): 319-333. doi: 10.1016/j.cytogfr.2013.01.005.

[ 10 ] Reits E A，Hodge J W，Herberts C A， et al. Radiation modulates the peptide repertoire, enhances MHC class I expression, and induces successful antitumor immunotherapy[J]. J Exp Med，2006，203 (5): 1259-1271. doi: 10.1084/jem.20052494.

[ 11 ] De Ruysscher D，Reynders K，Van Limbergen E， et al. Radiotherapy in combination with immune checkpoint inhibitors[J]. Curr Opin Oncol，2017，29 (2): 105-111. doi: 10.1097/CCO.0000000000000352.

[ 12 ] Bernstein M B，Krishnan S，Hodge J W， et al. Immunotherapy and stereotactic ablative radiotherapy (ISABR): a curative approach?[J]. Nat Rev Clin Oncol，2016，13 (8): 516-524. doi: 10.1038/nrclinonc.2016.30.

[ 13 ] Hiniker S M，Reddy S A，Maecker H T， et al. A Prospective Clinical Trial Combining Radiation Therapy With Systemic Immunotherapy in Metastatic Melanoma[J]. Int J Radiat Oncol Biol Phys，2016，96 (3): 578-588. doi: 10.1016/j.ijrobp.2016.07.005.

[ 14 ] Marconi R，Strolin S，Bossi G， et al. A meta-analysis of the abscopal effect in preclinical models: Is the biologically effective dose a relevant physical trigger?[J]. PLoS One，2017，12 (2): e0171559. doi: 10.1371/journal.pone.0171559.

[ 15 ] Britschgi C，Riesterer O，Burger I A， et al. Report of an abscopal effect induced by stereotactic body radiotherapy and nivolumab in a patient with metastatic non-small cell lung cancer[J]. Radiat Oncol，2018，13 (1): 102. doi: 10.1186/s13014-018-1049-3.

[ 16 ] Xu M J，Wu S，Daud A I， et al. In-field and abscopal response after short-course radiation therapy in patients with metastatic Merkel cell carcinoma progressing on PD-1 checkpoint blockade: a case series[J]. J Immunother Cancer，2018，6 (1): 43. doi: 10.1186/s40425-018-0352-8.

[ 17 ] Fehrenbacher L，Spira A，Ballinger M， et al. Atezolizumab versus docetaxel for patients with previously treated non-small-cell lung cancer (POPLAR): a multicentre, open-label, phase 2 randomised controlled trial[J]. Lancet，2016，387 (10030): 1837-1846. doi: 10.1016/s0140-6736(16)00587-0.

[ 18 ] Borghaei H，Paz-Ares L，Horn L， et al. Nivolumab versus Docetaxel in Advanced Nonsquamous Non-Small-Cell Lung Cancer[J]. N Engl J Med，2015，373 (17): 1627-1639. doi: 10.1056/NEJMoa1507643.

[ 19 ] Brahmer J，Reckamp K L，Baas P， et al. Nivolumab versus Docetaxel in Advanced Squamous-Cell Non-Small-Cell Lung Cancer[J]. N Engl J Med，2015，373 (2): 123-135. doi: 10.1056/NEJMoa1504627.

[ 20 ] Antonia S J，Villegas A，Daniel D， et al. Durvalumab after Chemoradiotherapy in Stage III Non–Small-Cell Lung Cancer[J]. New England Journal of Medicine，2017，377 (20): 1919-1929. doi: 10.1056/NEJMoa1709937.

[ 21 ] Garon E B，Rizvi N A，Hui R， et al. Pembrolizumab for the treatment of non-small-cell lung cancer[J]. N Engl J Med，2015，372 (21): 2018-2028. doi: 10.1056/NEJMoa1501824.

[ 22 ] Herbst R S，Baas P，Perez-Gracia J L， et al. PD1.06 (also presented as P2.41): Pembrolizumab vs Docetaxel for Previously Treated NSCLC (KEYNOTE-010): Archival vs New Tumor Samples for PD-L1 Assessment[J]. J Thorac Oncol，2016，11 (10s): S174-s175. doi: 10.1016/j.jtho.2016.08.014.

[ 23 ] Dranoff G，Jaffee E，Lazenby A， et al. Vaccination with irradiated tumor cells engineered to secrete murine granulocyte-macrophage colony-stimulating factor stimulates potent, specific, and long-lasting anti-tumor immunity[J]. Proc Natl Acad Sci U S A，1993，90 (8): 3539-3543. doi:

[ 24 ] Golden E B，Chhabra A，Chachoua A， et al. Local radiotherapy and granulocyte-macrophage colony-stimulating factor to generate abscopal responses in patients with metastatic solid tumours: a proof-of-principle trial[J]. The Lancet Oncology，2015，16 (7): 795-803. doi: 10.1016/s1470-2045(15)00054-6.

[ 25 ] Deng G，Hu P，Zhang J， et al. Elevated serum granulocyte-macrophage colony-stimulating factor levels during radiotherapy predict favorable outcomes in lung and esophageal cancer[J]. Oncotarget，2016，7 (51): 85142-85150. doi: 10.18632/oncotarget.13202.

[ 26 ] Benedict S H，Yenice K M，Followill D， et al. Stereotactic body radiation therapy: the report of AAPM Task Group 101[J]. Med Phys，2010，37 (8): 4078-4101. doi: 10.1118/1.3438081.
